# Characterizing mood disorders in the AFFECT study: a large, longitudinal, and phenotypically rich genetic cohort in the US

**DOI:** 10.1038/s41398-022-01877-2

**Published:** 2022-03-25

**Authors:** Maria Dalby, Morana Vitezic, Niels Plath, Lene Hammer-Helmich, Yunxuan Jiang, Chao Tian, Devika Dhamija, Catherine H. Wilson, David Hinds, 23andMe Research Team, Stella Aslibekyan, Adam Auton, Elizabeth Babalola, Robert K. Bell, Jessica Bielenberg, Katarzyna Bryc, Emily Bullis, Daniella Coker, Gabriel Cuellar Partida, Sayantan Das, Sarah L. Elson, Teresa Filshtein, Kipper Fletez-Brant, Pierre Fontanillas, Will Freyman, Anna Faaborg, Shirin T. Fuller, Pooja M. Gandhi, Julie M. Granka, Karl Heilbron, Alejandro Hernandez, Barry Hicks, Ethan M. Jewett, Katelyn Kukar, Keng-Han Lin, Maya Lowe, Jey C. McCreight, Matthew H. McIntyre, Steven J. Micheletti, Meghan E. Moreno, Joanna L. Mountain, Priyanka Nandakumar, Elizabeth S. Noblin, Jared O’Connell, Yunru Huang, Joanne S. Kim, Vanessa Lane, Aaron A. Petrakovitz, G. David Poznik, Morgan Schumacher, Anjali J. Shastri, Janie F. Shelton, Jingchunzi Shi, Suyash Shringarpure, Christophe Toukam Tchakouté, Vinh Tran, Joyce Y. Tung, Xin Wang, Wei Wang, Peter Wilton, Corinna Wong, Patrick F. Sullivan, Joshua W. Buckholtz, Jordan W. Smoller

**Affiliations:** 1 H. Lundbeck A/S, Valby, Denmark; 2 Department of Medical Epidemiology and Biostatistics, Karolinska Institutete, Stockholm, Sweden; 3 23andMe Inc, Sunnyvale, CA USA; 4 Department of Genetics and Psychiatry, University of North Carolina at Chapel Hill, Chapel Hill, NC USA; 5 Department of Psychology, Harvard University, Cambridge, MA USA; 6 Department of Psychiatry, Massachusetts General Hospital, Boston, MA USA; 7 Stanley Center for Psychiatric Research, Broad Institute of MIT and Harvard, Cambridge, MA USA; 8 Psychiatric and Neurodevelopmental Genetics Unit, Massachusetts General Hospital, Boston, MA USA

**Keywords:** Depression, Bipolar disorder, Clinical genetics

## Abstract

There has recently been marked progress in identifying genetic risk factors for major depression (MD) and bipolar disorder (BD); however, few systematic efforts have been made to elucidate heterogeneity that exists within and across these diagnostic taxa. *The Affective disorders, Environment, and Cognitive Trait (AFFECT) study* presents an opportunity to identify and associate the structure of cognition and symptom-level domains across the mood disorder spectrum in a prospective study from a diverse US population.

Participants were recruited from the 23andMe, Inc research participant database and through social media; self-reported diagnosis of MD or BD by a medical professional and medication status data were used to enrich for mood-disorder cases. Remote assessments were used to acquire an extensive range of phenotypes, including mood state, transdiagnostic symptom severity, task-based measures of cognition, environmental exposures, personality traits. In this paper we describe the study design, and the demographic and clinical characteristics of the cohort. In addition we report genetic ancestry, SNP heritability, and genetic correlations with other large cohorts of mood disorders.

A total of 48,467 participants were enrolled: 14,768 with MD, 9864 with BD, and 23,835 controls. Upon enrollment, 47% of participants with MD and 27% with BD indicated being in an active mood episode. Cases reported early ages of onset (mean = 13.2 and 14.3 years for MD and BD, respectively), and high levels of recurrence (78.6% and 84.9% with >5 episodes), psychotherapy, and psychotropic medication use. SNP heritability on the liability scale for the ascertained MD participants (0.19–0.21) was consistent with the high level of disease severity in this cohort, while BD heritability estimates (0.16–0.22) were comparable to reports in other large scale genomic studies of mood disorders. Genetic correlations between the AFFECT cohort and other large-scale cohorts were high for MD but not for BD. By incorporating transdiagnostic symptom assessments, repeated measures, and genomic data, the AFFECT study represents a unique resource for dissecting the structure of mood disorders across multiple levels of analysis. In addition, the fully remote nature of the study provides valuable insights for future virtual and decentralized clinical trials within mood disorders.

## Introduction

Mood disorders have a high lifetime prevalence in the general population and represent the leading cause of disability worldwide [1, 2]. Moreover, mood disorders cause marked impairment in social and occupational functioning, resulting in a high burden for the individual and to society [3, 4]. Twin and family studies show moderate-high heritability for these syndromes, indicating a prominent role for genetic variation in conferring susceptibility [5–8]. MD has a lifetime prevalence of 15% [9] and twin-heritability of 30–40% [5, 10]. In contrast, BD has a lifetime prevalence of 2.4% [11] and twin-heritability ~70% [6, 12]. Genomic analyses have shown that mood disorders are highly polygenic with likely thousands of small-effect loci contributing to susceptibility [13, 14]. Significant progress has been made in identifying common genetic risk variants associated with MD and BD, most recently from the Psychiatric Genomics Consortium (PGC). The PGC Bipolar working group identified 40 independent BD loci in a sample of 40,000 BD cases [15], and the PGC MD working group identified 102 independent loci associated with MD from more than 246,000 cases [16]. Despite these successes, a major obstacle in psychiatric genetics is our inability to map these signals to the symptom patterns, cognitive deficits and maladaptive decision-making that characterize mood disorders.

One critical open question is how genetic risk affects human cognition to predispose the development of mood disorder symptoms and related behaviors. With up to 90% of patients with major depression (MD) or bipolar disorder (BD) exhibiting impairment in multiple domains of cognition, this represents an important diagnostic and symptomatic feature in mood disorders and a key determinant of functional recovery [17, 18]. Much of the morbidity and mortality in mood disorders is due to behavioral factors, such as substance abuse, aggression, self-harm, and risky sexual behavior [19–21]. These behaviors, in turn, are thought to result from deficits in cognitive processes related to cost-benefit decision-making, reinforcement learning, social cognition, and executive function [22]. Many groups have reported phenotypic associations between mood disorders and some of these cognitive processes [23, 24]. However, such studies are typically small in size, limited in scope, and genetically uninformative, limiting insight into the underlying causes of cognitive dysfunction and maladaptive behavior in mood disorders.

It is widely recognized that the DSM-based nosology of psychiatric illness poorly captures two important features of mental disorders: the high degree of comorbidity between diagnostic taxa, and the profound symptom-level heterogeneity that exists within a given diagnostic taxon [22, 25–27]. These features suggest the existence of latent transdiagnostic symptom clusters in mood disorders and are consistent with evidence for shared genetic liability between otherwise categorically distinct psychiatric disorders [28–33]. To date, we know little about how much of the shared variance among mood disorder symptoms, cognitive function and maladaptive behavior is due to genetic factors. Likewise, GWAS estimate the proportion of variance in liability attributable to common variants genome-wide (SNP-heritability) to be ~9% for MD and 18% for BD [15], which are fractions of the pedigree-based estimated heritability. This accords with the significant role of non-genetic factors in mood disorder risk. In particular, a number of environmental risk factors have been identified for mood disorders, including poverty and traumatic life events, particularly in early life. Understanding the mechanisms through which such environmental influences interact with genetic susceptibility is key to elucidating the risk architecture of mood disorders. However, existent GWAS data sets are unable to answer these and other important open questions because of practical constraints that preclude the collection of an appropriately rich set of phenotypic data at scale.

To bridge these gaps, we leveraged technological advances in web-based participant recruitment, diagnostic assessment and cognitive testing to create the AFFECT study. The AFFECT study employed a longitudinal case-control design in nearly 50,000 US-based participants with BD, MD, and controls. Study participants were recruited from the 23andMe, Inc research participant database and through social media, representing a diverse sample that includes patients who may be underrepresented in clinical samples. A key innovation of this study is the depth of phenotypic data acquired, made practical through the use of online data collection. The study collected 9 months of remote phenotypic assessments, including recent and lifetime diagnostic evaluations, transdiagnostic symptom assessments, longitudinal measures of symptom state severity, and detailed medication profiling. Further, we obtained detailed information about environmental risk and protective factors, personality traits, and real-world maladaptive behaviors related to mood disorder morbidity and mortality. Finally, we measured task-based cognitive performance using an online testing battery. In this paper, we present the AFFECT study design, enrollment process, data collection, and characterize the MD, BD, and control groups based on baseline descriptive characteristics and genetic analysis. Lastly, we assess cohort representativeness and disorder severity and demonstrate the similarity of the case groups to those from prior large-scale genomic studies.

## Methods

### Cohort design

This genetic, case-control study was designed to enroll three cohorts: 15,000 participants with MD, 10,000 participants with BD, and 25,000 controls with no lifetime MD or BD. Of these, 1533 participants (3.06%) withdrew consent or failed to return the spit kit or intake survey before the study termination date and were excluded.

Participant eligibility criteria were: age between 18 and 50 years upon enrolment; residence in the United States; access to a desktop or laptop computer; and no reported diagnosis of Parkinson’s disease, essential tremor, schizophrenia, or Alzheimer’s disease. Enrollment required that the participants self-reported having been diagnosed with MD or BD by a medical professional and prescribed medication to treat such a disorder. Enrollment into the control cohort required that participants reported no lifetime diagnosis of BD, MD, generalized anxiety disorder, or post-traumatic stress disorder (PTSD) as well as never having been prescribed an antidepressant, mood stabilizer, or antipsychotic medication. All study participants had to provide informed consent and a saliva sample for SNP array genotyping, and be willing to complete the online study sessions over the course of 9 months.

**Fig. 1:**
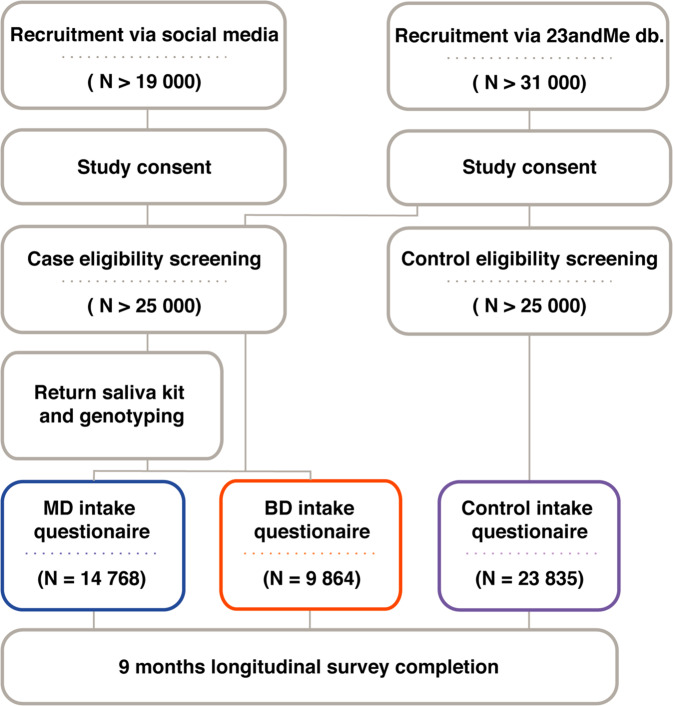
Flowchart illustrating the enrollment. The procedural steps were: Informed consent, apply for enrollment and meet study inclusion and no exclusion criteria, return a saliva kit for genotyping (except for excisting costumers who purchased and returned a 23andMe kit prior to joining the study), and answer the baseline questionnaire. In the 9 months after enrolment, participants were asked to complete monthly surveys and cognitive tests.

The study was conducted between August 2017 and September 2019 and online recruitment of participants, genotyping, and survey data collection were performed by 23andMe. Figure 1 illustrates the enrollment flow and study setup*.* Participants were recruited through two channels: all controls and approximately one-fifth (*n* = 4997) of all case participants were recruited from 23andMe’s existing customer database through email or logged-in website invitation. All other case participants (*n* = 9635) were recruited through social media such as Facebook and enrolled as new 23andMe customers. Study participants who met the eligibility criteria received compensation depending on if they were existing or new 23andMe customers. Existing customers, who had purchased a 23andMe kit prior to joining the study, received a $20 Amazon gift card. New customers received the 23andMe^®^ Health + Ancestry Service, including a DNA test kit, at no cost.

### Study assessments

The study content was designed by the AFFECT investigators and administered by 23andMe. The self-reported survey and test battery (Table 1) was initiated at session 1 with an extensive *background survey* covering: demographics (i.e., age, gender, race, ethnicity), socioeconomic information (i.e., marital status, current employment, education, parental education, income), clinical details about the given disorder (cases only; e.g., age of onset, current and past episode characterization), family psychiatric history, the Self-rated Diagnostic and Statistical Manual of Mental Disorders (DSM-5) Level 1 Cross-Cutting Symptom Measure, and adverse childhood experiences (scale and scoring details in Supplementary Materials).

**Table 1:** Study content overview.

Sessions	1	2	3	4	5	6	7	8	9
Demographic and clinical surveys									
Background survey									
DSM-5 Level 1 - cross-cutting [44]									
Adverse Childhood Experiences [48]									
Demographic, Socio-economic information	x								
Lifetime disorders information									
Family mental health history									
Symptom State and Medication									
ASR-Mania [64]									
PROMIS-Depression [65]									
Medication history, current use, recent changes	x	x	x	x	x		x		x
Recent activities (e.g. smoking, sleep habits)									
Childhood Exposure To Abuse and Household Dysfunction Questionaire [47]						x			
Perceived social support [66]								x	
Behavioral assessments									
Risky, Impulsive, and Self-destructive behavior Questionnaire (RISQ) [67]						x			
Self-Reported Psychopathy (SRP-SF) [68]								x	
Cognitive assessments									
Digit-Symbol Substitution Test (DSST) [69]	x								
Probability-, and Delay-Discounting Task (PD/DD) [70–73]									x
Gradual Onset Continuous Performance Test (Grad-CPT) [74]			x						x
THINC-Integrated Tool (THINC-it) [75] (incl. Perceived Deficits Questionnaire for Depression-5-item (PDQ-5-D) [76]		x					x		
Balloon Analog Risk Task (BART) [77]				x					x
Reading the mind in the eyes (RMET) [78]					x				

The table displays the study battery, showing the timing of all assessments. Participants had 1 month window for completion of assessments for a given session, thus session 1 = month 0 (baseline), Session 2 = month 1, and so forth.

The *mood and medication survey* was also given at session 1 and repeated in sessions 2–5, 7, and 9. This survey included: medication history (session 1), changes in medications (all follow-up surveys), life events/life style (e.g., alcohol use and sleep patterns), Altman Self-rating of Mania (ASRM) scale, and Patient-Reported Outcomes Measurement Information System (PROMIS)-Depression scale (scale and scoring details in supplementary materials). The study battery further included standardized behavioral tasks assessing risk, impulsivity and psychopathic traits and five cognitive tools designed to assess different domains of functioning. The cognitive tests were either given at one or two time points as noted in Table 1.

### SNP genotyping

We evaluated common variant genetic contributions to risk for MD and BD using SNP array data. DNA extraction and genotyping were performed on saliva samples by the National Genetics Institute, a CLIA-licensed clinical laboratory and a subsidiary of the Laboratory Corporation of America. Samples were genotyped, phased and imputed by 23andMe standardized pipeline, as described in detail in Supplementary Methods. Roughly 9.22 million high-quality genotyped and imputed SNPs on autosomal and X chromosome were tested.

For each GWAS, we restrict participants to a set of individuals who had a specified ancestry determined through an analysis of local ancestry estimation [34] and a maximal set of unrelated individuals was chosen for each GWAS analysis using a segmental identity-by-descent (IBD) estimation algorithm [35].

### Genome-wide associations

GWAS was performed on MD versus controls, BD versus controls, mood disorder (MD, BD) versus controls and MD versus BD using a logistic regression model: *case/control ~ age* + *sex* + *top 5 Principal Components (PCs) + genotyping platforms* + *genotype*. GWAS was first performed separately on individuals of European, African American, East Asian, Latino ancestry, and combined by fixed-effect meta-analysis using METAL [36]. GWAS results were adjusted for the genomic control inflation factors, which can be found under each Manhattan plot in Supplementary Figures. Note that the study enrollment channel (existing/enrolled customers) was embedded in the genotype platform term, where around 80% of existing customers were genotyped on 23andMe’s genotype platform v4, while all newly enrolled participants were genotyped on platform v5 (Supplementary Table 1). Across all results, we removed SNPs that had an available sample size of less than 20% of the total GWAS sample size; where logistic regression results that did not converge due to complete separation, identified by absolute value of effect size or standard error greater than 10 on the log-odds scale; or that had MAF < 0.1%.

### SNP-heritability and genetic correlations

We used LD score regression (LDSC) [37] v1.0.1 to estimate SNP-heritability ($h_{SNP}^2$) from GWAS summary statistics for European ancestry MD and BD including variants with *r*^2^ > 0.8 and minor allele frequency ≥0.01. Estimates of $h_{SNP}^2$ on the liability scale depend on the assumed lifetime prevalence of each disorder in the population (K). We report $h_{SNP}^2$ with K = [0.001–0.3] for MD and K = [0.001–0.03] for BD.

Genetic correlations (r_g_) to external summary statistics were also performed using LDSC [37]. External data included; the PGC MDD meta-analysis samples PGC-MDD1 (2013) [10], PGC-MDD2 excluding the 23andMe sample (2018) [38], and PGC-MDD3 excluding the 23andMe sample (2019) [16]; the 23andMe discovery sample of MDD (herein Hyde et. al, 2016; where 5.0% of MD cases and 4.3% of controls from the AFFECT study were also included in Hyde et al.) [39]; the two most recent PGC BD meta-analysis samples PGC-BD2 (2019) [40] and PGC-BD3 (including the PGC-BD3 type I and type II sub-cohorts (2020) [15]); the most recent PGC SCZ meta-analysis samples PGC-SCZ2 (2014) [41] and PGC-SCZ3 (2020) [41, 42]. Data was obtained from https://www.med.unc.edu/pgc/download-results/ and through the 23andMe data-access portal.

### Statistical analyses

Sample comparisons were conducted using R (v3.5.2). Descriptive statistics were performed on the total participation pool and on subgroups: the three cohorts of MD, BD, and controls; within subtype of BD diagnosis (BD1 vs. BD2) and, within each cohort subgroups based on enrollment strategy (i.e., participants drawn from the 23andMe database and participants enrolled through social media for this study). For categorical variables, the number and percentage were reported for each value. For quantitative variables, the mean, median, standard deviation, and ranges were reported. Differences in demographic and clinical covariates were compared using regression models (continuous or categorical variables) and Fisher’s exact tests for categorical variables.

## Results

### Cohort characteristics

A total of 48,467 participants were included in these analyses: 14,768 reported that they had been diagnosed and treated for MD, 9864 had been diagnosed and treated for BD, and 23,835 were controls with no lifetime history of MD or BD (Fig. 1). The BD cohort contained 3070 (31.2%) BD subtype I (BD1), 5053 (51.3%) BD subtype II (BD2), and 1718 (17.5%) did not specify the latest type of BD diagnosis received (BD unspecified-type). Among all participants, 72% were female and the mean age was 32.3 years (range 18–52 years). Most participants were of European ancestry (71.9%) followed by Latino (14.2%), African American (3.8%), and East Asian (3.6%) ancestry (Table 2).

**Table 2:** Demographics features of all study participants and mood disorder cases and controls seperately.

	Total (*N* = 48,467)	Control (*N* = 23,835)	MD (*N* = 14,768)	BD (*N* = 9864)
Age (years)				
Mean (SD)	32.3 (8.0)	32.7 (8.2)	31.7 (7.7)	32.1 (7.8)
Median	31	32	31	31
Q1, Q3	26, 38	26, 39	26, 37	26, 37
Sex, *n*, (%)			12,067 (81.7)	8002 (81.1)
Female	34,986 (72.2)	14,917 (62.6)		
Broad ancestry, *n* (%)				
African American	1850 (3.8)	1037 (4.4)	453 (3.1)	360 (3.6)
European	34,863 (71.9)	15,680 (65.8)	11,570 (78.3)	7613 (77.2)
Latino or Hispanic	6886 (14.2)	4041 (17.0)	1665 (11.3)	1180 (12.0)
East Asian	1734 (3.6)	1450 (6.1)	209 (1.4)	75 (0.8)
Other	3134 (6.5)	1.627 (6.8)	871 (5.9)	636 (6.4)

Broad ancestry groups are genetically estimated.

Participant completion rates ranged from 28 to 100% (mean 42.6%) per session and were lower for cognitive assessments than for surveys (Supplementary Table 2). Study retention (i.e., number of assessments completed) was highest for MD cases (mean 50.2%, SD 30.6) followed by BD cases (mean 45.2%, SD 30.6), lowest for controls (mean 38.2%, SD 28.8), and higher in females (mean 45.0%, SD 30.1) than males (mean 38.7%, SD 29.9) (Supplementary Fig. 1). Study retention was positively correlated with educational level and age, and negatively correlated with reported adverse childhood experience score, BMI, ASRM score, and the DSM-5 cross-cutting domains of substance use, anxiety, depression, anger, suicidal ideation, and sleep problems (Supplementary Fig. 2).

Marital status, highest education achieved, and current socioeconomic status were reported at baseline and followed-up by a brief status assessment during each longitudinal assessment. Overall, socioeconomic status was significantly lower for cases, especially BD participants (Supplementary Table 3). In particular, we found that 19.5% and 26.9% of MD and BD participants, respectively, were currently not in paid employment as compared to only 7.3% of the control cohort. We observed an ascertainment effect in which case participants drawn from the 23andMe database (existing consumers) showed higher yearly salary and educational level than those enrolled through social media (multivariate analysis, *P* < 1.0 × 10^−16^). After adjusting for enrollment method, however, significant socioeconomic differences remained between cases and controls (multivariate analysis, *P* < 1.0 × 10^−16^, Supplementary Table 3).

### Disease history

**Fig. 2:**
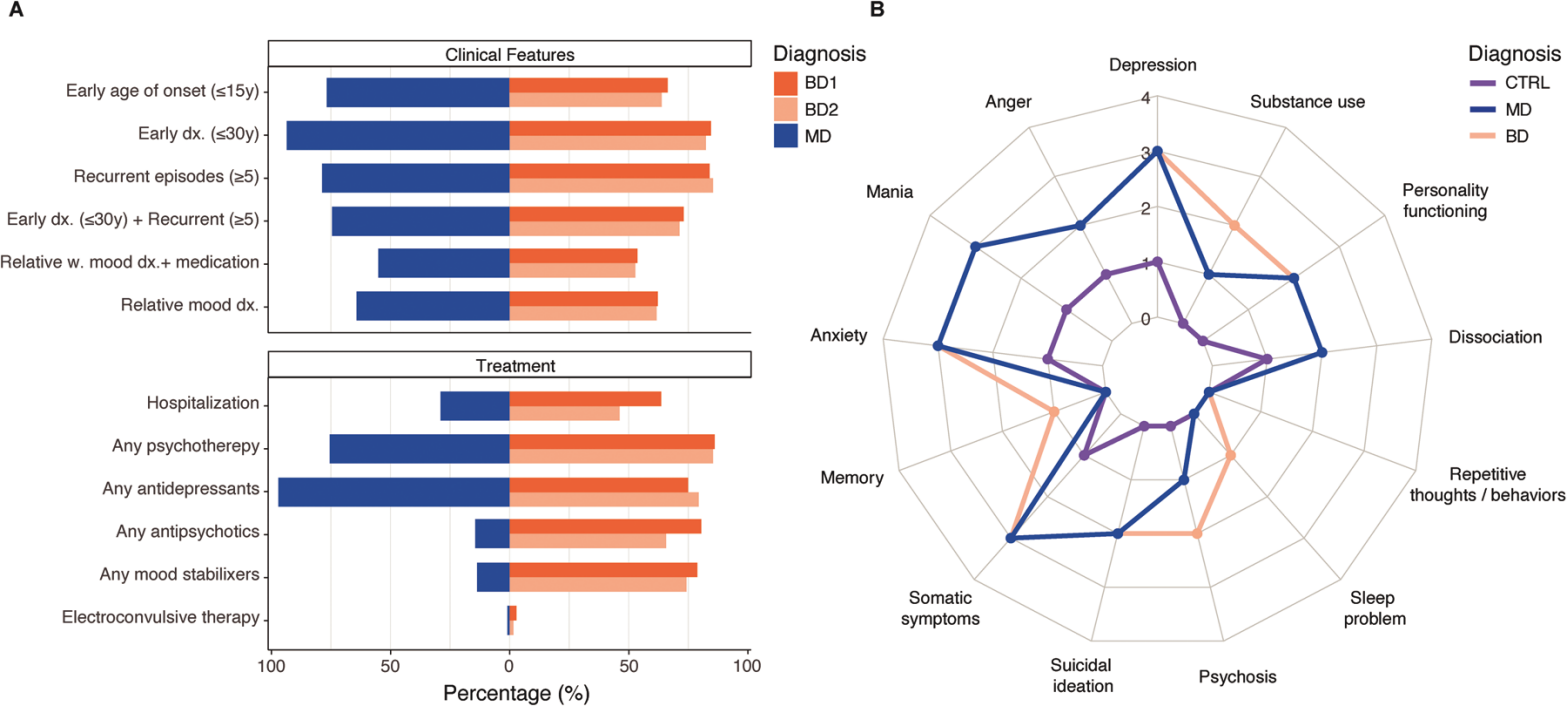
Baseline clinical features. **A** Summary of key clinical features in cases reporting a diagnosis of MDD, BD subtype 1 (BD1) and BD subtype 2 (BD2), as per latest diagnosis recieved. Mood disorders cases in this 23andMe sub-cohort show high burdens of illness. Any medication class refers to medication received over the last 5 years and during the study. Percentage of those who answered one or several treatment questions in the medication survey. **B** Transdiagnostic symptoms. Radar plot of median score pr. symptom domain within controls (purple), MD (blue), and BD (orange) participants. Scores are based on the DSM-5 cross-cutting symptom measures, where max item score (ranging from 0 to 4) within each domain is reported and summarized.

Figure 2A and Supplementary Table 4 summarizes the clinical features of MD and BD cases and highlights that both MD and BD presented with high disease severity. Most participants reported symptom onset in adolescence (MD; mean 13.2 (SD 5.1), BD; mean 14.3 (SD 5.2)) while formal psychiatric diagnosis was not typically received until early adulthood (MD; mean 19.5 (SD 6.6), BD; mean 23.2 (SD 7.6)), consistent with prior studies [43–45]. The course of illness differed between the disorders; BD cases tended to report short but recurrent episodes: 52.2% of the participants had experienced >10 episodes and 80.0% reported a typical episode duration of <3 months. In contrast, MD cases had fewer episodes of longer duration: 59.7% had experienced ≤10 episodes, 47.7% reported a typical episode duration of 3–6 months or longer, and 10.0% reported episode duration ≥1 year (Fig. 2A, Supplementary Fig. 3A, B).

As expected, psychotropic medication use was common, since this was an inclusion criterion: 23,202 (96.4%) of cases reported having taken medication for a mood disorder in the prior 5 years, 17,292 (70.2 %) were taking medication at baseline, and 7726 (31.4%) began or restarted a medication during the study. MD and BD participants (respectively) reported use of the following treatments in the prior 5 years and/or at present: antidepressants 13,803 (95.4%) and 8508 (88.1%); mood stabilizers 4875 (33,8%) and 8394 (86.9%); antipsychotics 6133 (42.4%) and 7972 (82.5%); and electroconvulsive therapy 107 (0.9%) and 144 (2.0%). Most cases had received cognitive or behavioral psychotherapy in the past 5 years (MD 9770, 67.7%, BD 7235, 79.3%; Supplementary Fig. 3C, D), most commonly 1–2 times a week. BD1 cases had the highest rates of symptom-related hospitalization (63.6%), although the rates were also high for the other mood disorder diagnoses (BD2, 46.1%; MD, 29.0%).

### Symptom state

Nearly half of the MD cases (*N* = 6971, 47.5%) and about a quarter of the BD cases (2729, 27.8%) reported that they were experiencing an episode at baseline (Table 3, Supplementary Fig. 4). Most BD participants reported their current episode as depressive (1694, 62.7%). A current manic episode was reported in 219 (7.13%) BD1 participants, 106 (6.17%) unspecified-type BD participants, and a current hypomanic episode was reported across BD type: BD1 140 (16.0%), BD2 407 (29.4%), and 55 (11.9%) unspecified-type. We further observed that participants enrolled through social media exhibite greater disease burden (Supplementary Fig. 5, Supplementary Table 4) and were more likely to be in active mood episode compared to participants drawn from the 23andMe research participant database (41.0% versus 34.0%).

**Table 3:** Self-identified episode (i.e. “Are you currently experiencing an episode?”, “What type of episode are you experiencing?”) and symptom scale-based episode of cases at baseline.

	Self-identified episode (A), *n* (%)	Symptom-scale episode (B), *n* (%)	Self-identified and Symptom-scale episode (A∩B), *n* (% A | B, % B|A)	Cohen’s κ
MD				
Depressive	6971 (47.5)^a^	8575 (59.3)^b^	5980 (87.5, 70.1)	0.43 (±0.01)
BD				
Depressive	1694 (17.3)^c^	5502 (57.0)^d^	1637 (93.5, 71.4)	0.36 (±0.04)
Manic/hypomanic	927 (9.5)^c^	2754 (28.5)^d^	613 (66,7, 22.4)	0.22 (±0.02)

The intersection (A∩B) shows number and proportion overlap, where A|B: self-report given symptom-based outcome, B|A: symptom-based outcome given self-report. Cohen’s κ given with 0.05 confidence interval (CI). *N* based on ^a^ = 14,447, ^b^ = 14,690, ^c^ = 9663, ^d^ = 9802.

We defined probable depressive episodes using the Level 1 DSM-5 cross-cutting measure—depressive domain (score ≥ 2) and the PROMIS-depression scale (*T*-score ≥60), which identified 71.7% of all cases being in a depressive episode at baseline. Additionally, we defined a probable manic/hypomanic episode from the Level 1 DSM-5 cross-cutting measure—manic domain (score ≥ 2) and the ASRM scale (score > 5), identifying 28.5% of BD participants being in an episode at baseline (Table 3). When comparing the symptom scale-based episodes with the self-identified episodes at baseline, we found reasonable correspondence for depressive episodes (κ = 0.43 and κ = 0.36 respectively for MD and BD) and a more modest correspondence for manic or hypomanic episodes (κ = 0.22).

### Symptom-level comorbidities

The DSM-5 self-rated cross-cutting symptom measure assesses 13 transdiagnostic symptom domains of relevance across psychiatric diagnosis [34] (scoring details given in Supplementary Materials). We found that both MD and BD participants exhibited a wide-range of transdiagnostic symptoms (median number of positively screened symptom domains = 9), a clear distinction to the control cohort (median number of positively screened symptom domains = 2) (Fig. 2B, Supplementary Table 5). The most common symptom domains in cases were depression, mania, somatic symptoms (i.e. aches and pains), and anxiety. Furthermore, sleep problems and substance use symptoms provided the strongest differentiation of BD from MD (multivariable analysis, coefficient 0.43 (95% CI ±0.03) *P* < 2.2e^−16^, coefficient 0.41 (95% CI ±0.05) *P* < 2.2e^−16^, respectively).

Regarding non-psychiatric conditions, MD and BD participants reported higher rates of comorbidities compared to controls. This was particularly evident for inflammatory and neurological disorders (multivariable analysis, OR ≥ 3.03 *P* < 0.001, Supplementary Table 3).

### Family psychiatric history

Family history prevalence of anxiety disorder, MD, BD, or PTSD in first-degree relatives is shown in Supplementary Table 6. Rates were significantly higher for all disorders among cases (78.4 %) compared to controls (Fisher’s exact OR = 4.2 (95% CI ±0.1), *P* < 2.2e^−16^), particularly for the same disorder and within BD subtypes (Fisher’s exact OR (95% CI) MD = 6.6 (0.6), BD1 = 3.1 (±0.4), OR = 5.0 (±1.0), *P* < 2.2e^−16^, Supplementary Fig. 6). The prevalence of mental disorders in first-degree relatives of controls (33.0%) was comparable to rates reported in population-based samples [46].

### Environmental influences

Reported adverse childhood experiences (ACE) were assessed across multiple domains (i.e., psychological and sexual abuse, neglect, and household dysfunction) [47, 48]. Childhood adversity was common, with 63.9% of participants reporting at least one ACE. The total ACE score was significantly higher in cases than controls, with almost twice as many ACEs reported (case mean = 3.96, control mean = 2.00, *P* < 1.0 × 10^−16^). Moreover, BD cases reported more ACEs than MD cases (Supplementary Table 7). Within ACE domains, physical and emotional neglect showed the largest association with mood disorders (OR = 5.6, 95% CI ±0.4); again, these associations were considerably stronger in BD cases (OR = 6.54, 95% CI ±0.34).

### SNP-heritability and genetic comparability

GWAS was conducted in European ancestry participants for mood disorder (MD + BD), each disorder separately, BD subtypes, and comparing MD versus BD. Furthermore, a trans-ethnic meta-analysis of European, Latino, African American and East Asian GWAS was conducted for MD and for BD. Variant-level analysis, which was not the focus of this paper, is provided in Supplementary Figs. 7–22 and sample sizes for each GWAS can be found in Supplementary Table 8.

**Fig. 3:**
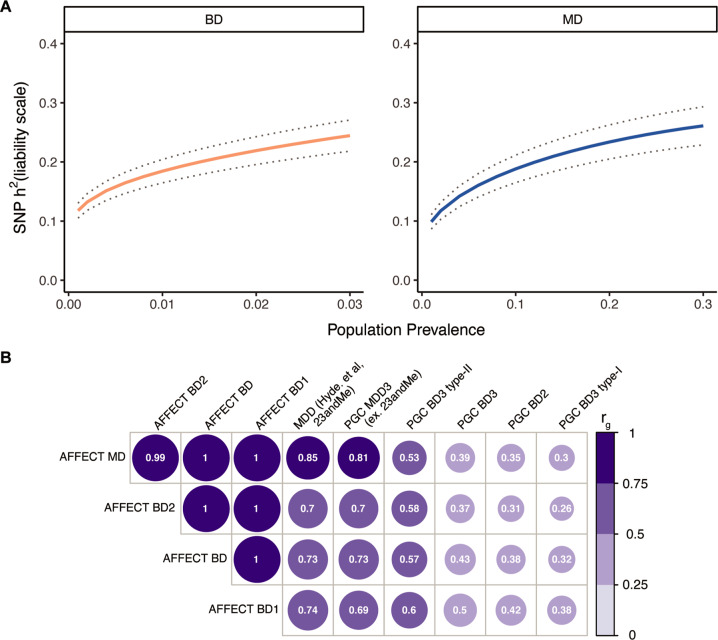
Genetic component. **A** Liability-scale SNP-heritability of AFFECT BD and MD as a function of population prevalence, ranging from 0.001 to 0.03 for BD and 0.001–0.3 for MD with r_g_ estimates at every 0.001 step-wise increase. Dotted line represents s.e. **B** Estimated genetic correlations of European ancestry AFFECT BD and MD with PGC GWAS of MDD3 (excluding the 23andMe cohort), the 23andMe MD discovery cohort (Hyde et al, 2016), PGC-BD2, and of PGC-BD3, which is further divided into BD3 type I and type II. Correlations in AFFECT BD were performed with the full cohort (BD) and within BD type (BD1, BD2). All correlations were significant, circle size and values indicate r_g_. *P*-values, *Z*-scores and s.e are reported in Supplementary Table 9.

The SNP-heritability ($h_{SNP}^2$) on the liability scale for European ancestry MD was 0.19 (SE 0.02) and 0.21 (SE 0.03) for a population prevalence of 0.10 and 0.15, respectively. These estimates are higher than those reported in previous self-reported or broadly ascertained MD cohorts [39, 49]. The SNP-heritability for European ancestry BD was comparable to previous large cohorts [1, 15, 40] with $h_{SNP}^2$ estimates of 0.16 (SE 0.02) and 0.22 (SE 0.02) on the liability scale assuming population prevalence of 0.005 and 0.02, respectively (Fig. 3A).

To further compare the MD and BD cohorts to other mood disorder studies, we estimated genetic correlations (r_g_) to the most recent and largest meta-analysis samples (Fig. 3B, Supplementary Table 9). We found that r_g_ for AFFECT-MD was highest with PGC MD2 (0.85 (SE 0,06), *P* = 2.1 × 10^−40^), followed by significant correlations to the other MD cohorts, then PGC BD2 type II. We found significant, but moderate, genetic correlation between and the PGC3 BP cohort (0.43 (SE 0.04), *P* = 5.3 × 10^−22^). Of note, stronger genetic correlations were observed between the AFFECT-BD cohort and prior MD samples (0.61 (SE 0.1) – 0.78 (SE 0.08)), suggesting that the current BD cohort is genetically different than previously published BD cohorts that used more traditional clinical ascertainment (see Discussion). Genetic correlations of AFFECT-BD1 and BD2 cases to external data showed an increased positive correlation between BD1 and external BD cohorts (0.42 (SE 0.07) – 0.59 (SE 0.12)) and SCZ cohorts (0.30 (SE 0.07) – 0.33 (SE 0.06)), while the genetic correlations of BD2 was greater for external MD cohorts (0.46 (SE 0.1) – 0.71 (SE 0.06) and the PGC3 BP type II cohort (0.56 (SE 0.08), *P* = 1.2e−10).

## Discussion

The AFFECT study was initiated to advance our understanding of phenotypic and genetic heterogeneity in MD and BD and to clarify the role of shared genomic and environmental risk factors that may transcend their diagnostic boundaries. Several aspects of AFFECT are notable including the administration of task-based measures indexing multiple domains of cognition (e.g. executive, motivational, and social) that capture key facets of the Research Domain Criteria (RDoC) [50] framework; transdiagnostic symptom assays; the assessment of trait and environmental risk and resilience factors; and the repeated measures design enabling analysis of change in symptoms and multi-domain cognitive task performance. Here, we have presented baseline characterization of the cohort and summarized the clinical features of MD and BD cases.

The US-based study participants were ascertained from the general 23andMe participant database and from social media. Control participants did not self-report diagnosis of or treatment for mood disorders. Case participants self-reported a clinican-ascertained diagnosis of MDD or BD (I or II) and were currently using one or more prescribed medications to manage their symptoms. Additional study ascertainment criteria pertained to age (18–50 years old) and the absence of of Parkinsons disease, Alzheimers disease, essential tremor, or schizophrenia diagnosis. Demographic and socio-economic features of BD and MD cases in the AFFECT study were largely comparable to those reported in epidemiologic and clinical samples [51–53] with a substantial female predominance among cases. Consistent with prior research [54, 55], reported adverse childhood experiences were relatively common and associated with significantly increased risk of mood disorder.

Prior studies have shown that selective participation represents a potential source of bias in both epidemiological and genetic association studies [56, 57]. Consistent with this, several features of the cohort differ from those seen in many clinically ascertained mood disorder cohorts. For example, educational attainment and income levels among MD cases were somewhat higher than reported in population-based samples [52] as might be expected given the ascertainment through a direct-to-consumer genomics company. Interestingly, we observed some differences within the sample: lower socioeconomic status and greater illness severity were observed among those recruited through social media compared to participants drawn from the existing 23andMe consumer database. Although it might be expected that cases recruited through direct-to-consumer genomics and social media platforms would have less burden of illness compared with those ascertained clinically, this was not the case. In fact, most mood disorder cases in this study reported early-onset illness, recurrent episodes, positive family history, and treatment with medication and psychotherapy. Indeed, a history of psychiatric hospitalization among MD cases was higher (29%) than that reported in a representative sample of US adults (12%) [52]. Together, these suggest a high disease burden (significant impairment and dysfunction) in our cohort.

Overall, 71.7% of AFFECT participants reported symptoms of a current depressive episode at baseline, and 28.1% of BD cases reported current manic or hypomanic symptoms. This likely reflects the fact that BD2 was overrepresented in our BD cohort (51.3%) relative to population-based samples [11, 53], but may also suggest that remote study participation is more likely for euthymic and depressive BD patients. We found that the agreement between self-reported and mood scale ratings for mania was limited. This underlines the limitations of self-reported assessments and symptom-based outcomes as discussed elsewhere [58].

Despite these considerations, we expect the AFFECT study to contribute importantly to understanding the genetic basis of mood disorders. The incorporation of transdiagnostic symptom and behavior measures, longitudinal symptom assessments, and task-based measures of neuro- and social cognition, make this a unique resource for genomic studies. In the initial GWAS of the AFFECT mood disorders, we identified several genome-wide significant loci; the strongest association was between MD and SNPs within *NEGR1*, a gene encoding a synaptic adhesion protein that has been robustly associated with depression in prior studies [16, 59]. Recent analyses have found that GWAS of MD samples characterized by “minimal phenotyping” (e.g. based on self-report of prior diagnosis and/or treatment for depression) show lower heritability and are enriched for less specific genetic effects on MD compared with samples diagnosed using strict syndromal criteria [60]. In this context, it is notable that the estimated liability scale *h*^*2*^_*SNP*_ for AFFECT MD (0.19–0.21) is in the same range as “strictly-defined lifetime MDD” in that analysis and higher than what is seen in broadly-defined MD cohorts, including the previous 23andMe self-reported depression cohort [16, 38, 39]. As demonstrated in previous work [61, 62], SNP heritability is a consequence of several known and unknown effects, including the exclusion of specific comorbidities, disease severity, and the use of controls from which other psychiatric disorders have been excluded [63].

Genetic correlation analyses indicate that AFFECT MD is highly correlated (r_g_ = 0.71–0.85) with MD ascertained in studies included in the PGC. Unexpectedly, however, genetic correlations between AFFECT-BD and published PGC GWAS of BD were relatively modest (r_g_s = 0.38–0.43) while the genetic correlation between the AFFECT MD and AFFECT BD was approximating 1. Indeed, the pattern of genetic correlations seen with AFFECT-BD closely resembled those of AFFECT-MD and did not vary substantially by AFFECT-BD subtype 1 or 2. While recent genetic studies have shown that depression and bipolar depression have a large genetic overlap and many symptoms co-occur [17], we speculate that study exclusion of comorbid SCZ diagnosis and the fully remote ascertainment and follow-up strategy might have affected study participation, e.g deselected BD cases with psychotic features. Furthermore, the high genetic correlation within the AFFECT study sub-cohorts may have been affected by the use of fully shared controls that were screened for both MD and BD (i.e. “extreme” controls). Together, these results suggest a large genetic overlap with depression and high variability between different BD samples, further underlining the importance of understanding heterogeneity within and across diagnostic taxa.

The AFFECT study represents a unique cohort of remotely recruited individuals with MD and BD and controls. The availability of repeated measures over time as well as task-based cognitive domains will provide an important opportunity to examine the genomic basis of mood disorders and underlying traits. More in-depth analyses of these phenotypes and shared or unique contributions to BD and MD are forthcoming.

## Supplementary information

Supplementary Materials

Supplementary Tables 1-9

Supplementary Table 10

Supplementary Table 11

Supplementary Table 12

Supplementary Table 13

Supplementary Table 14

Supplementary Table 15

## Data Availability

The top 10,000 SNPs for each GWAS are provided in Supplementary Tables 10–15. Participants provided informed consent and participated in the research online, under a protocol approved by the external AAHRPP-accredited IRB, Ethical & Independent Review Services (E&I Review). Participants were included in the analysis on the basis of consent status as checked at the time data analyses were initiated. The full GWAS summary statistics for the 23andMe discovery data set will be made available through 23andMe to qualified researchers under an agreement with 23andMe that protects the privacy of the 23andMe participants. Please visit https://research.23andme.com/collaborate/#dataset-access/ for more information and to apply for access. Individual-level data are not publicly available due to participant confidentiality, and in accordance with the IRB-approved protocol under which the study was conducted. Researchers interested in the study’s individual-level data may apply to the 23andMe Research Innovation Collaborations program.
