# Supplementary material for: Characterizing mood disorders in the AFFECT study: a large, longitudinal, and phenotypically rich genetic cohort in the US
Source: Transl Psychiatry. 2022 Mar 25;12:121. doi: 10.1038/s41398-022-01877-2 (PMC8956583; doi:10.1038/s41398-022-01877-2)
Supplement: Supplementary file 1 — Supplementary Materials [file 41398_2022_1877_MOESM1_ESM.docx]

Characterizing Mood Disorders in the AFFECT study: A Large, Longitudinal, and Phenotypically Rich Genetic Cohort in the US

Supplementary methods

**Standardized assessments and scoring**

DSM-5 Level 1 Cross-Cutting Symptom Measure – Adult^1^. This self-report assessment consists of 23 questions assessing the presence and severity of 13 transdiagnostic symptom domains in the prior two weeks: depression, anger, mania, anxiety, somatic symptoms, suicidal ideation, psychosis, sleep problems, memory, repetitive thoughts/behaviors, dissociation, personality functioning, and substance use. Each item is rated on a 5-point scale (0=none or not at all; 1=slight or rare, less than a day or two; 2=mild or several days; 3=moderate or more than half the days; and 4=severe or nearly every day. According to the measurement scoring manual, a rating of mild (i.e., 2) or greater on any item within a domain (except for substance use, suicidal ideation, and psychosis) is considered a positive screening outcome. For substance use, suicidal ideation, and psychosis, a rating of slight (i.e., 1) or greater on any item within the domain serve as a positive screen.

Altman Self-rating of Mania (ASRM) Scale^2^: This self-report scale evaluates the severity of core mania symptoms in the prior week: elevated/euphoric mood, increased self-esteem, decreased need for sleep, pressured speech, and psychomotor agitation. Each item is rated on a 5-point scale (0 to 4) summed to obtain a total score ranging from 0–20. A total score of ≥6 indicates a high probability of mania or hypomania. If 4 of the 5 items were answered, a prorated score was calculated. The ARSM was chosen because it has excellent test-retest reliability, construct validity, is sensitive to state change, and has a low subject burden^2,3^.

Patient-Reported Outcomes Measurement Information System (PROMIS) Depression scale^4^. This self-rating measure assesses depressive symptoms by 8 items during the past week. Each item is rated on a 5-point scale (1=never; 2=rarely; 3=sometimes; 4=often; and 5=always) and summed to obtain a total score ranging from 8 to 40. If 6 or 7 items were answered, a prorated score was calculated. The total score was transformed into a T-score using the APA conversion table (PROMIS Health Organization and PROMIS Cooperative Group.). The T-scores were interpreted as follow: <50 none to slight, 55-59.9 mild, 60-69.9 moderate, and ≥70 severe depression. The PROMIS Depression scale was chosen based on high test-retest reliability in remote settings, is sensitive to state change, and has a low subject burden^5^.

The ASRM and the PROMIS-depression scale have been selected by the American Psychiatric Association as “DSM-5 Level 2 Cross-Cutting Symptom Measures” of mania and depression symptom domains identified in the DSM-5 Level 1 Cross-Cutting Symptom Measure. For reference, a DSM-5 level 1 depression domain score ≥2 is considered a threshold for “mild or greater” depressive symptoms. PROMIS Depression T-score ≥60 indicate mild to severe depressive episode. A DSM-5 level 1 mania domain score ≥2 is considered a threshold for “mild or greater” mania symptoms. A ASRM scores of > 5 indicates a probable manic or hypomanic episode^1^.

Childhood Exposure To Abuse and Household Dysfunction Questionaire^6^ consists of 17 items that measure 10 categories of exposure (prior to a respondent’s 18^th^ birthday) to adverse childhood experiences (ACEs): abuse (psychological, physical, and sexual); neglect (physical or emotional); and household dysfunction (witnessing violence (or threat of violence) directed towards maternal caregiver; living with household members who suffered from mental illness (including substance use disorders), attempted suicide, or were imprisoned)^6^. Perticipants were defined as exposed to a category if they responded “yes” to one or more of the questions in that category and the prevalence of positive responses to ACEs were assessed. In addition, the total sum of exposure is referred to as the ACE score and ranges from 0-10. If missing data was present, the particular ACE category and the total score were droped for that individual.

**Medication survey**

Past five years and current treatment was assessed at baseline (mood_medication_survey 1) with a follow-up questionnaire on changes in medication throughout the study (mood_medication_survey 2-7). Mood-stabilisers included Eskalith® or Lithobid® / lithium, Depakote® or Depakene® / valproate or divalproex, Tegretol® / carbamazepine, Trileptal® / oxcarbazepine, Lamictal® / lamotrigine, another mood-stabilizer. Antipsychotic medications included Zyprexa® / olanzapine, Seroquel® / quetiapine, Risperdal® / risperidone, Abilify® / aripiprazole, Geodon® / ziprasidone, Latuda® / lurasidone, Rexulti® / brexpiprazole, another antipsychotic medication. Antidepressents included Celexa® / citalopram, Lexapro® / escitalopram, Prozac® / fluoxetine, Paxil® / paroxetine, Zoloft® / sertraline, Viibryd® / vilazodone, Luvox® / fluvoxamine, Trintellix® or Brintellix® / vortioxetine.

**SNP genotyping and imputation**

Samples were genotyped on one of five versions of the genotyping platforms. Platforms v1 and v2 had 566,870, and 573,100 SNPs, respectively, and were variants of the Illumina HumanHap550+ BeadChip with ~25K custom SNPs selected by 23andMe. Platform v3 has 955,616 SNPs and is based on the Illumina OmniExpress+ BeadChip with custom content to improve overlap with the v2 array. Platform v4 is a customized array of 596,632 SNPs, including a subset of v2 and v3 SNPs with additional coverage of lower-frequency coding variation. Platform v5 is an Illumina Infinium Global Screening Array (~640,000 SNPs) supplemented with ~50,000 SNPs of custom content. Samples with < 98.5% call rate were reanalyzed. Individuals whose analyses failed repeatedly were re-contacted by 23andMe to provide a new saliva sample. Samples genotyped on platforms v1-v4 were phased using Finch (ref), a modified version of Beagle^7^, to accommodate genotyping error and recombination. Samples from platform v5 were phased using Eagle2^8^. For imputation, a combined reference panel consisting of the 1000 Genomes phase 3 (May 2015 release) and the UK10K imputation were used. For each chromosome, the phased samples were imputed against the panel using Minimac3, where each chromosome of the reference panel was split into chunks of < 300,000 variants, with 10,000 overlapping variants on each side. Parameters were estimated using a single batch of 10,000 individuals for each chunk. Imputation of the HLA alleles was performed using HIBAG^9^ using default parameters. Imputed allelic dosage for *HLA-A, B, C, DPB1, DQA1, DQB1,* and *DRB1* were reported at four-digit resolution and translated into amino acid dosages using SNP2HLA^10^.

Genotyped SNPs were removed if they: were only genotyped on platforms v1 and v2 due to small sample size, failed a test of parent-offspring transmission based on parent-child trios ascertained from among 23andMe customers, were in Hardy-Weinberg P<10^-20^, had a call rate of <90%, had a genotype date batch effect P<10^-50^ by ANOVA, had a sex effect of r^2^>0.1 by ANOVA of SNP genotypes, or were matching multiple genomic positions in the reference genome. Imputed SNPs with a strong platform batch effect (P<10^−50^ by F test from an ANOVA of the SNP dosages against a factor representing v4 or v5 platform) were also removed.

Existing 23andMe customers were genotyped prior to this study, while case participants enrolled specifically for this study were all genotyped on the current v5 genotype array. Therefore, 23andMe genotype platform version and study enrolment channel (existing/newly enrolled customers) were essentially completely correlated in cases. The genotype platform was included in all GWAS as a covariate, thus also covering enrolment channel (Supplementary table 7).

**Ancestry determination**

For each GWAS, we restrict participants to a set of individuals who have a specified ancestry determined through an analysis of local ancestry. Briefly, the algorithm first partitions phased genomic data into short windows of about 300 SNPs. Within each window, we use a support vector machine (SVM) to classify individual haplotypes into one of 31 reference populations (https://www.23andme.com/ancestry-composition-guide/). The SVM classifications are then fed into a hidden Markov model (HMM) that accounts for switch errors and incorrect assignments and gives probabilities for each reference population in each window. Finally, simulated admixed individuals were used to recalibrate the HMM probabilities so that the reported assignments are consistent with the simulated admixture proportions. The reference population data is derived from public datasets (the Human Genome Diversity Project, HapMap, and 1000 Genomes), as well as 23andMe customers who have reported having four grandparents from the same country.

Ancestries are defined as follow:

| **Ancestry** | **Classification criteria** |
| --- | --- |
| European | European + Middle Eastern > 0.97, European > 0.90 |
| East Asian | East Asian + Southeast Asian > 0.97 |
| South Asian | South Asian > 0.97 |
| Middle Eastern (& North African) | Middle Eastern + European > 0.97, Middle Eastern > 0.90 |
| African American + Latinos | European + African + East Asian + Native American + Middle Eastern > 0.90, African + Native American > 0.01 |

African Americans and Latinos are admixed with broadly varying contributions from Europe, Africa and the Americas. Therefore, no single threshold of genome-wide ancestry will be able to effectively discriminate African Americans and Latinos. However, the distributions of the length of segments of European, African and American ancestry are very different between African Americans and Latinos, because of distinct admixture timing between the three ancestral populations in the two ethnic groups. Therefore, we trained a logistic classifier that takes one customer's length histogram of segments of African, European and American ancestry, and predict whether the customer is likely African American or Latino.

For GWAS, ancestry criteria classified participants into broad ancestries of European, African American, Latino, East Asian, South Asian, and Middle Eastern. For each ancestry, a principal component (PC) analysis was calculated using 65K high-quality genotyped SNPs present on all platforms for randomly sampled individuals across platforms. PC scores for participants not included in the analysis were obtained by projection. Finally, a maximal set of unrelated individuals was chosen for each GWAS analysis using a segmental identity-by-descent (IBD) estimation algorithm^11^. Individuals were defined as related if they shared more than 700 cM IBD (roughly 20% of the genome) including regions where the two individuals share either one or both genomic segments IBD.

**Supplementary Tables**

**Supplementary Table 1**

23andMe genotype platform (v2-v5) overview participants within diagnosis group (MD, BD) and further divided into enrollment method; participants drawn from the 23andMe research database (Existing) and participants enrolled through social media (Social media).

**Supplementary Table 2**

Survey completion rates (i.e. number of participants who have completed each assessment) given for all participants (Total) and within diagnosis group (Control, MD, BD).

**Supplementary Table 3**

Socioeconomic Status & Medical Conditions based on the Background Survey given in session 1. For survey questions with ordinal or interval variables, a cut-off was set to classify answers as dichotomous, as given in the header. Survey questions with multiple nominal variables were converted into boolean values for a given choice of answer. Regression analysis results of each dependent variable are given as odd-ratio (OR), 95% confidence interval (CI), and p-value, for a univariable analysis (explanatory variable: diagnosis group (control, MD, BD)), and multivariable analysis (explanatory variable: diagnosis group (control, MD, BD), enrolment channel (social media, existing db.)).

**Supplementary Table 4**

Disease history overview for MD and BD participants, based on the Background Survey given in session 1. Descriptive statistics are given for all case participants (Total), MD, BD, and in MD and BD split by enrollment method; participants drawn from the 23andMe reseaarch database (Existing) and enrolled through social media (Social media). Q1 and Q3 (lower- and upper-quantile), min (minimum), max (maximum), SD (standard deviation), n (number).

**Supplementary Table 5**

DSM-5 cross-cutting symptom scale descriptive statistics for all participants (Total) and within diagnosis group (Control, MD, BD). The maximum item score is reported within each symptom domain and the number of domains above the guided threshold (i.e. a positive screen) is given in “Number of domains above threshold”.

**Supplementary Table 6**

Disease history of first-degree relatives (mother, father, children, siblings) descriptive statistics for all participants (Total) and within diagnosis group (Control, MD, BD). Q1 and Q3 (lower- and upper-quantile), min (minimum), max (maximum), SD (standard deviation), n (number).

**Supplementary Table 7**

Adverse Childhood Event exposure based on the Childhood Exposure To Abuse and Household Dysfunction Questionaire. Exposure prevalence was defined as one or more positive responses within the listed categories. The ACE score was defined as the sum of ACE category exposures. Regression results of ACE score and categorical ACE domain exposure (dependent variables) are given as odd-ratio (OR), 95% confidence interval (CI), and p-value, for a univariable analysis (diagnosis group (control, MD, BD)), and multivariable analysis (including diagnosis group (control, MD, BD) and enrolment channel (Social media, Existing db.)).

**Supplementary Table 8**

Total number of participants included in each GWAS, i.e. each disorder versus controls, MD versus BD, and participants for mood disorder (MD+BD) versus controls. A trans-ethnic meta-analysis of European, Latino, African American and East Asian GWAS was conducted for MD and for BD.

**Supplementary Table 9**

LDSC genetic correlations reported for the AFFECT MD, AFFECT BD, AFFECT BD I and AFFECT BD II against external cohorts; PGC-MD1 (2013)^12^, PGC-MD2 (2018)^13^ and PGC-MD2 (2019)^14^ excluding the 23andMe sample, MDD 23andMe discovery (Hyde et. al)^15^, the two most recent PGC-BD including PGC-BD subtype cohorts (2019, 2020)^16,1717^, PGC-SCZ (2014, 2020)^18,19^. The columns represent: p1 = trait 1, p2 = trait 2, rg = genetic correlation, se = standard error of rg, p = p-value for rg; h2_obs, h2_obs_se = observed scale h2 for trait 2 and standard error, h2_int, h2_int_se = single-trait LD Score regression intercept for trait 2 and standard error, gcov_int, gcov_int_se = cross-trait LD Score regression intercept and standard error.

**Supplementary Table 10-15**

Summary statistics for top 10,000 most signficant SNPs in all European and cross-ancestry meta-analysis GWAS; Supplementary Table 10: MD vs control European; Supplementary Table 11: BD vs control European; Supplementary Table 12: Case (BD+MD) versus control European; Supplementary Table 13: MD versus BD European; Supplementary Table 14: MD versus control trans-ethnic meta-analysis; Supplementary Table 15: BD versus control trans-ethnic GWAS meta-analysis. All alleles are on the (+) or forward genomic strand. All effects are reported for the 2nd allele listed in alphabetical order. dose.b.0 = average dosage of the B allele in controls. avg.rsqr = average imputation r^2^ across all batches . Text representation of SNP location in relation to other genes in the region. The SNP location is denoted by []. If the SNP occurs between genes then the distance from those genes are denoted by dashes (-), with ‘’ = <1kb, ‘−‘ = <10kb, ‘−−‘ = <100kb, ‘−−−‘ = <1000kb. HG19 release of UCSC was used for mapping.

**Supplementary Figures**

**Supplementary Figure 1**

**A B**

*Study-completion rates. Percentage of study surveys and cognitive assessments completed pr. participant, given as the final number of assesments completed out of the total set of 21 assesments,, split by perticipant diagnosis label (****A****) and by sex (****B****).*

**Supplementary Figure 2**

**A B**


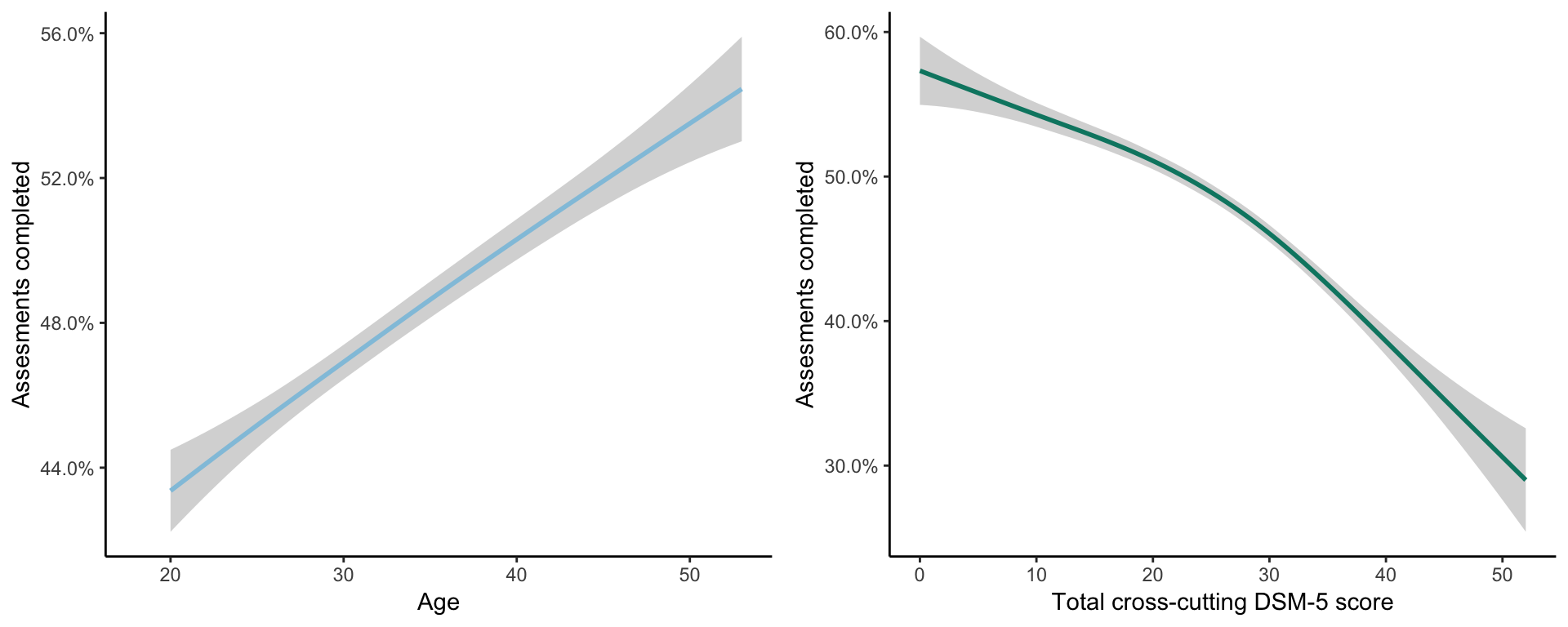

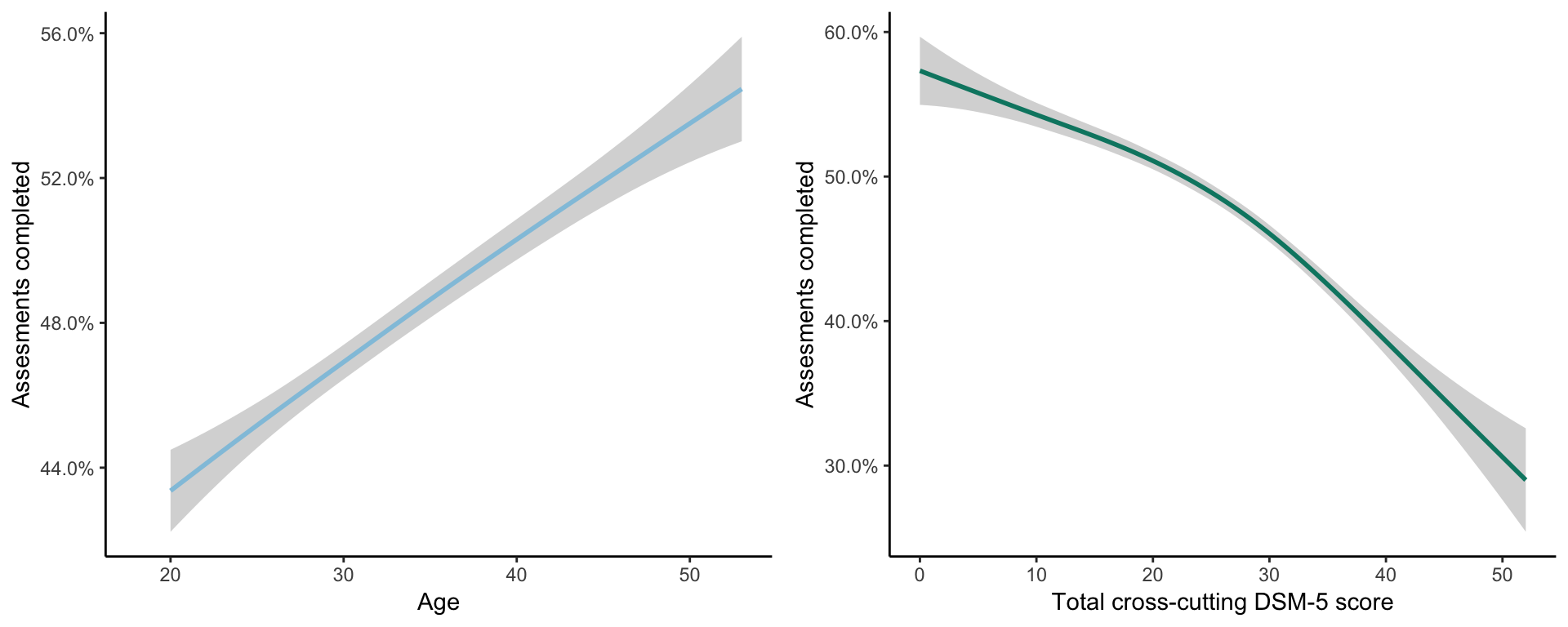


**C**

**
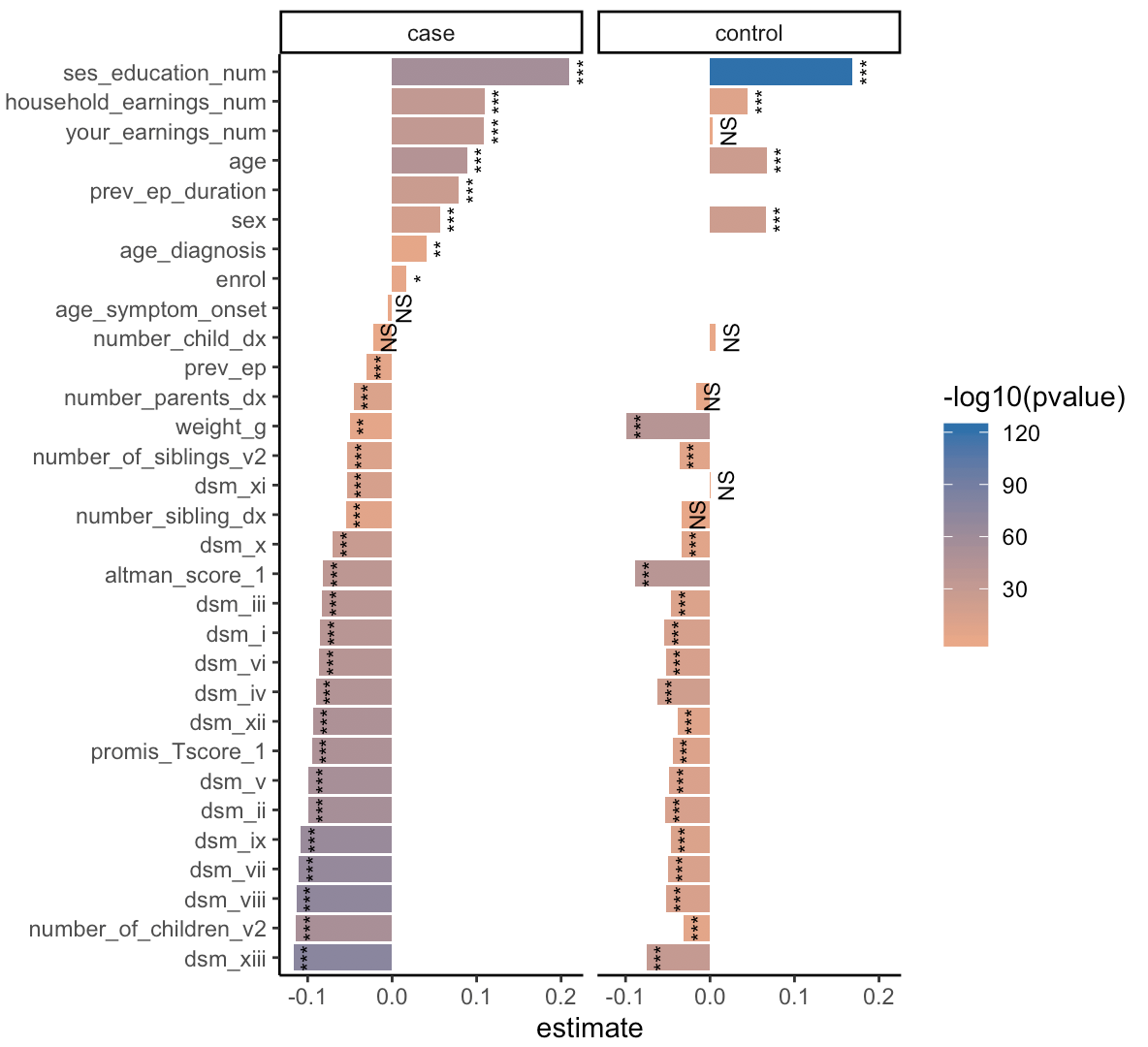
**

*Completion rates and correlations. Percentage of surveys and assessments completed pr. participant as a function of age (****A****) and DSM-5 sum score (****B****).* ***C****.* Correlations (Pearson's) *between completion rate and attributes given at baseline. Attributes (y-axis) are sorted according to correlation estimates r (x-axis)* of cases *and coloured according to significance level (-log10(P)).*

**Supplementary Figure 3**

**
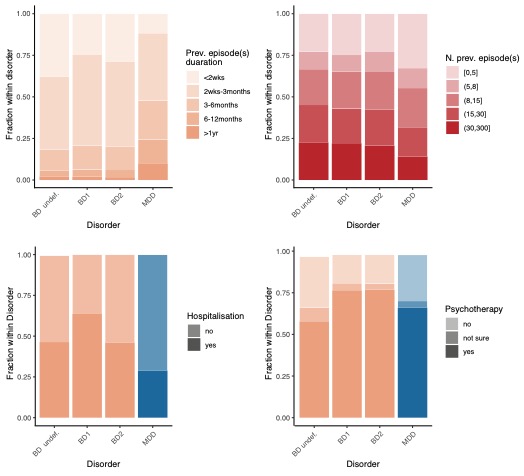
A B**

**C D**

**A.** *Fraction of reported typical duration of* previous episode(s). wks = weeks, yr = year. **B**. Fraction of binned number of previous episodes. Factions computed *within diagnosis subgroups (BD1, BD2, BD_undef (not reported) and MD subjects). Fraction of subjects ever being hospitalized (****C****) or having received psychotherapy within the last five years (****D****) within diagnosis subgroups (BD1, BD2, BD_undef (not reported or identified) and MD subjects).*

**Supplementary Figure 4**

**
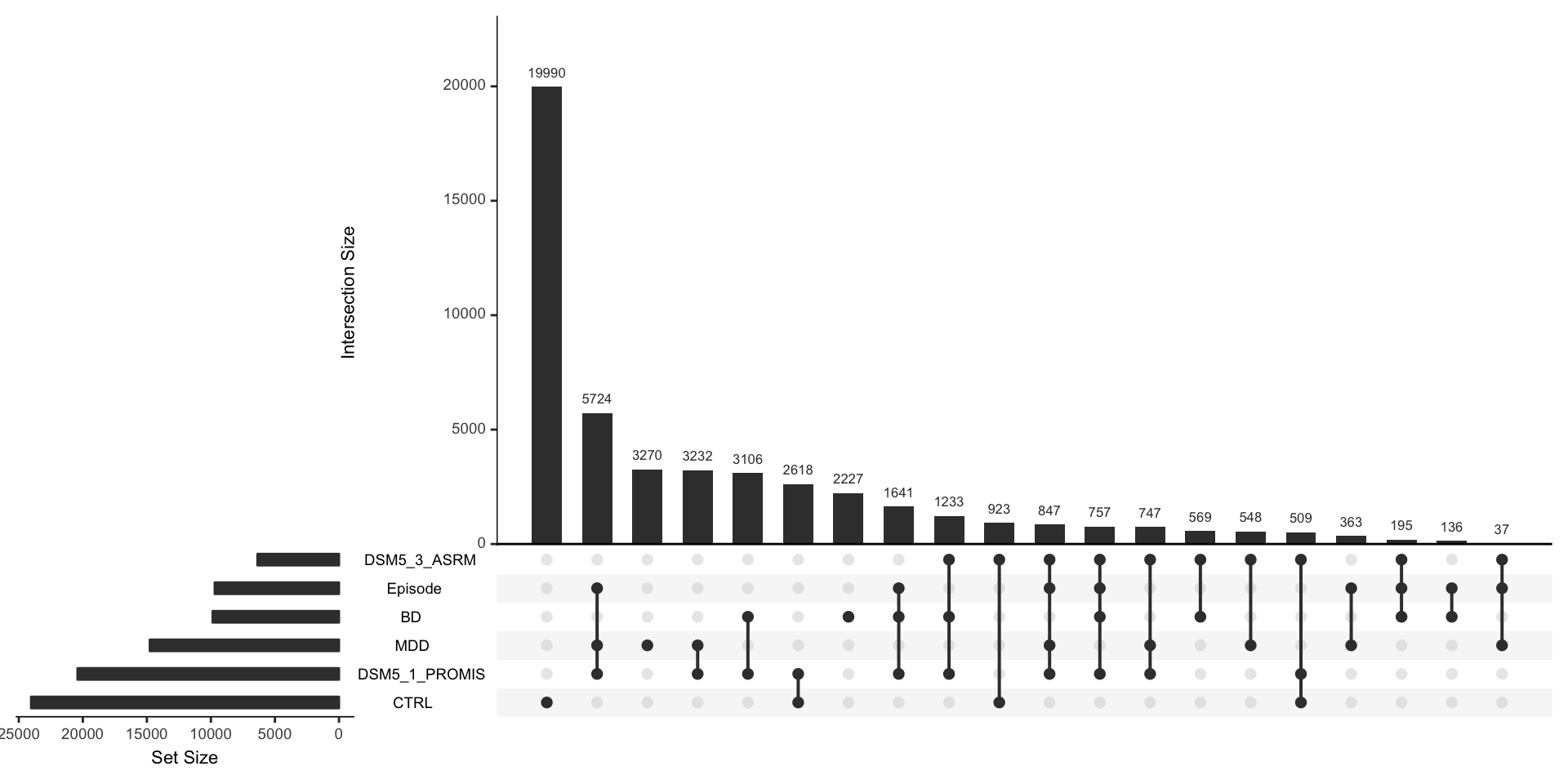
**

*Self-reported mood state at baseline assessments. Correspondence between subjective episode report (i.e. based on the questions;“Are you currently experiencing a depressive episode?” for MD and “Are you currently experiencing an episode?” for BD) and symptomatic scale-characterized episodes from the DSM-5 Depression domain and the PROMIS-depression scale (category DSM5_1_Promis) and from the DSM-5 Mania domain and the ASR mania-scale (category DSM5_3_ASRM). Left side: bar chart on number of subjects within each category. Right side: Bar plots on number of subjects combined from each category (upper). Category combination is shown by dot-plot (lower).*

**Supplementary Figure 5**


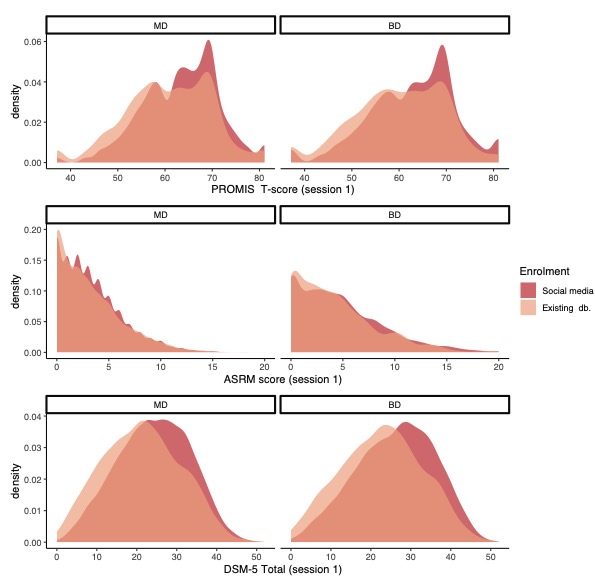


*Density plot of self-reported mood state at baseline (assessment one) in cases. First row: total PROMIS Depression T-score; second row: total ASRM score; third row: total DSM-5 cross-cutting score. Score destitutions are split by enrolment channel (red; participants enrolled through social media, orange; participants taken from the exciting 23andme database).*

**Supplementary Figure 6**

*First-degree relative reported mental condition, split by cohort. Percentage of subjects with a first-degree relative diagnosed with either PTSD, MDD, BD1, BD2, anxiety, or another mental illness (Other). Subjects are split by diagnosis cohort (BD, MD, and CTRL), the BD cohort is further split according to latest type of BD diagnosis received (light orange: not identified, orange: BD type 1, and red: BD type 2).*

**Supplementary Figure 7**


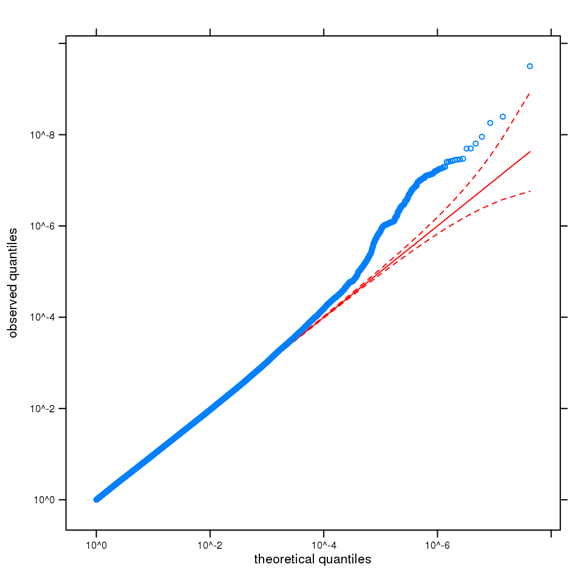


*MD versus control European* *Q-Q plot of observed versus expected quantiles for the GWAS P values, where the expected distribution of P values is uniform under the null hypothesis, plotted on a log scale. A solid red line is shown with a slope of 1, and dashed red lines represent a 95% confidence envelope under the assumption that the test results are independent. The test statistics in the Q-Q plot have already been adjusted for inflation.*

**Supplementary Figure 8**


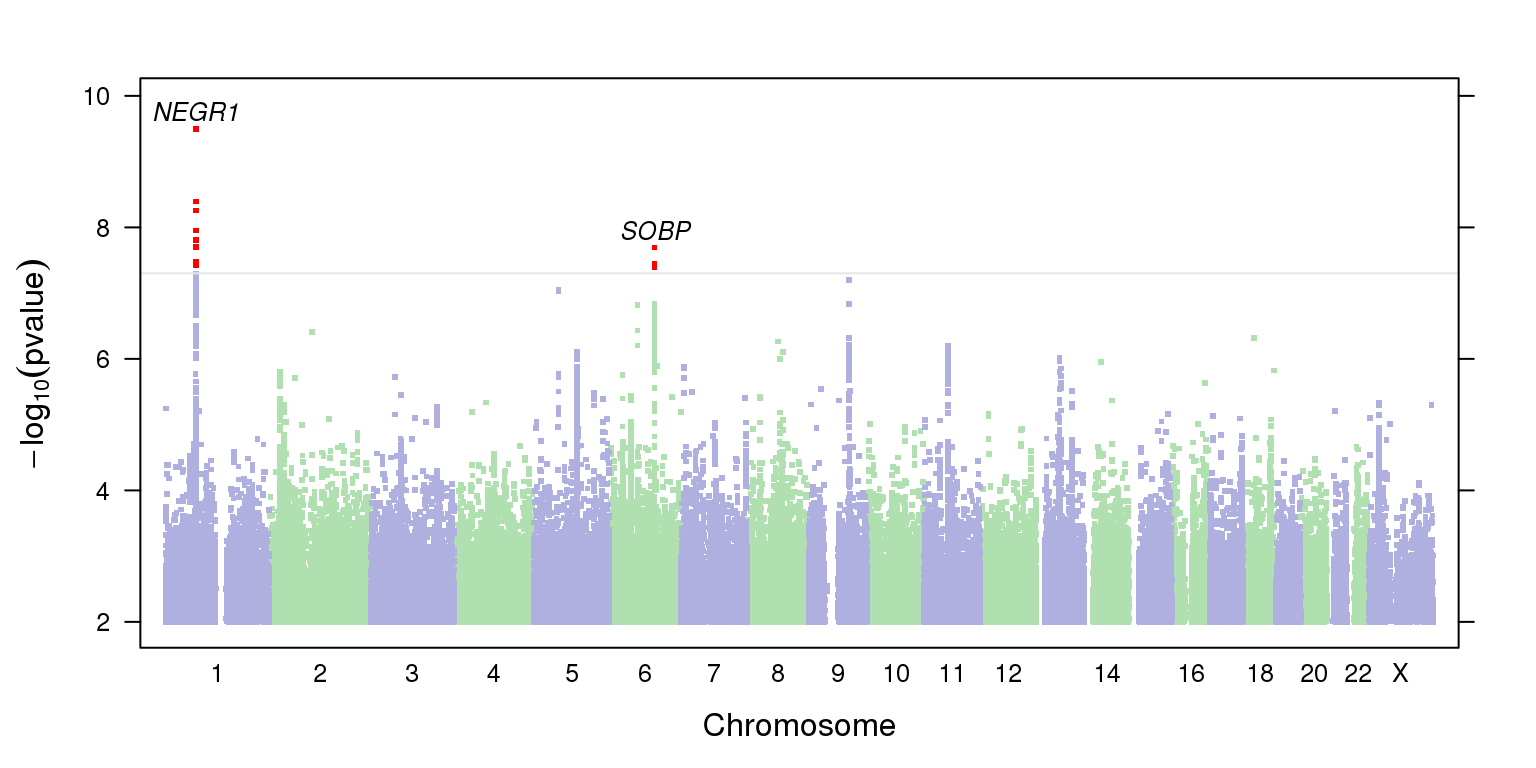
 *MD versus control European association Manhattan plot.* *Logistic regression assuming an additive model for allelic effects. Each SNP along the chromosome (x-axis) is plotted against the -log_10_(pvalue) test statistics*. *The results have been adjusted for a genomic control inflation factor****λ=1.064.***

**Supplementary Figure 9**

**A B**


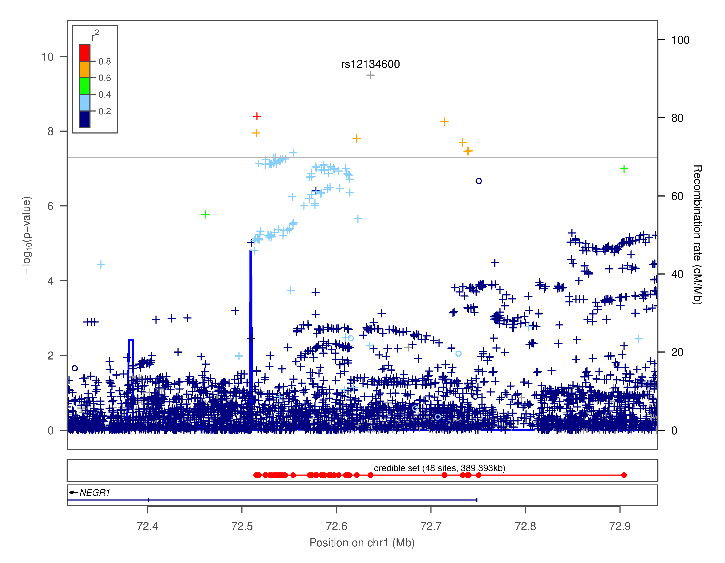

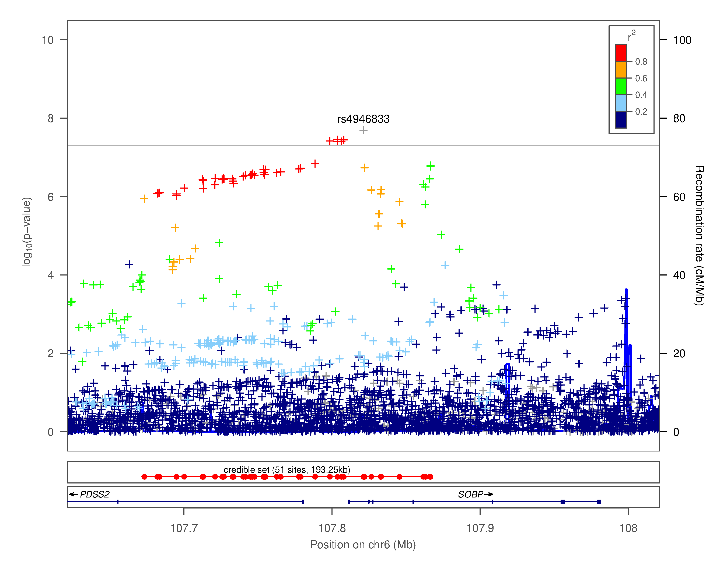


*MD versus control European regional plot for* ***A*** *rs12134600 [NEGR1],* ***B*** *rs4946833 [SOBP]. The regional association plots show association test statistics versus position in the vicinity of the strongest associations. In the plots, a ‘+’ indicates an imputed variant, whereas a ‘x’ indicates an imputed protein-altering variant. Likewise, an ‘o’ symbol indicates a genotyped variant and a ‘◇’ (diamond) indicates a genotyped protein-altering variant. For each association, we also report details of the credible set, calculated under the assumption of a single causal variant within the locus.*

**Supplementary Figure 10**


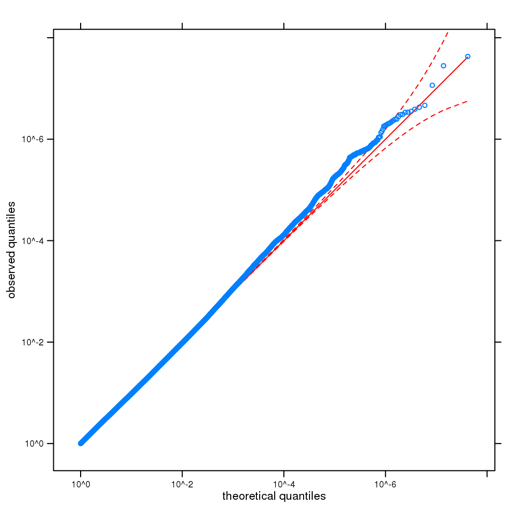


*BD versus control European* *Q-Q plot of observed versus expected quantiles for the GWAS P values, where the expected distribution of P values is uniform under the null hypothesis, plotted on a log scale. A solid red line is shown with a slope of 1, and dashed red lines represent a 95% confidence envelope under the assumption that the test results are independent. The test statistics in the Q-Q plot have already been adjusted for inflation.*

**Supplementary Figure 11**


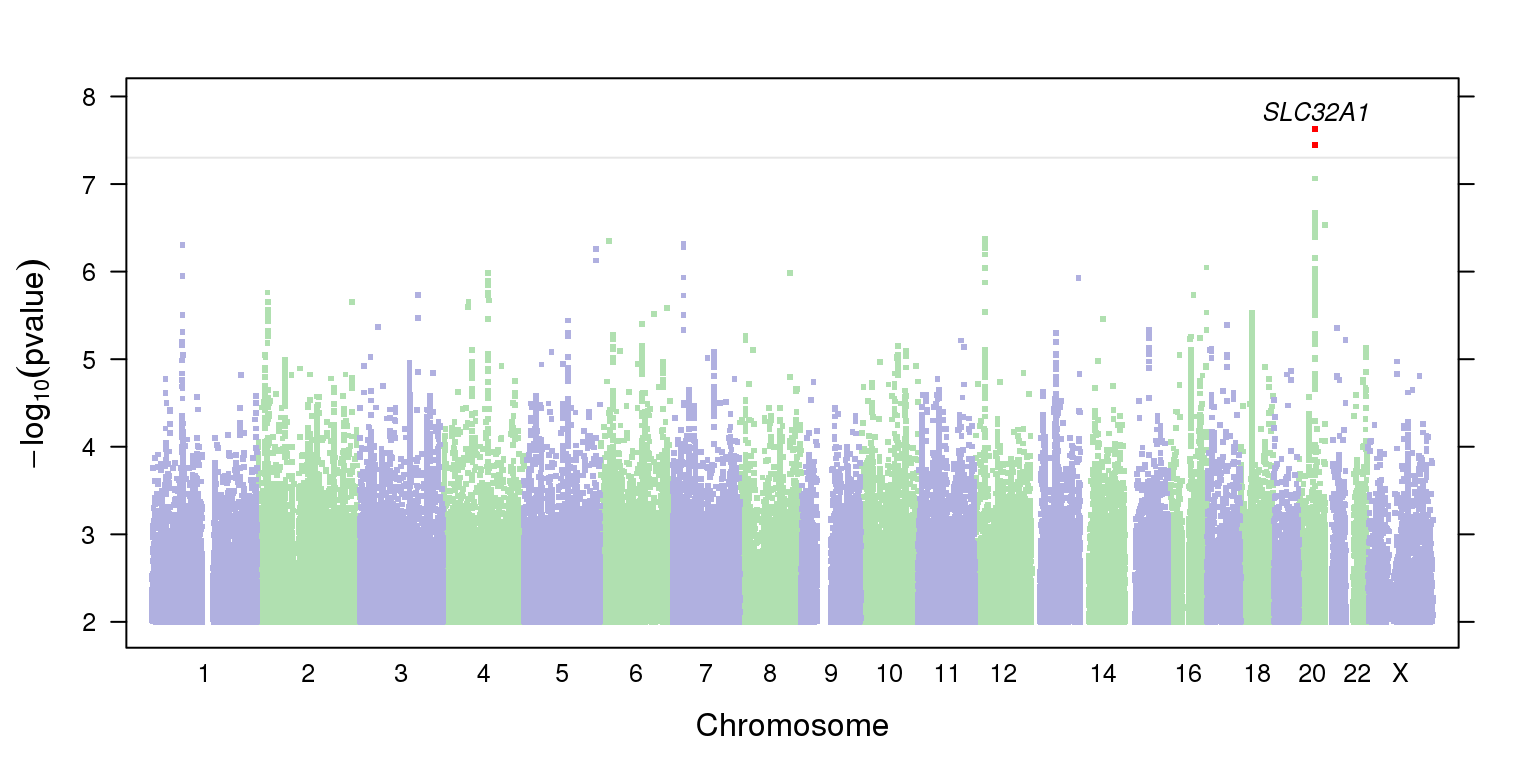


*BD versus control European association Manhattan plot.* *Logistic regression assuming an additive model for allelic effects. Each SNP along the chromosome (x-axis) is plotted against the -log_10_(pvalue) test statistics. The results have been adjusted for a genomic control inflation factor****λ=1.072.***

**Supplementary Figure 12**


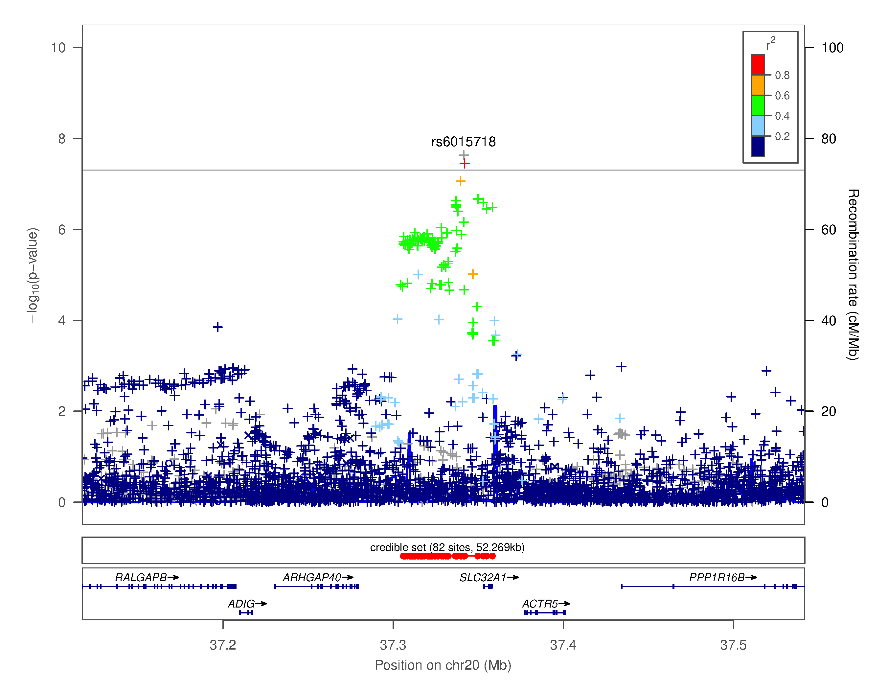


*BD versus control European regional plot for rs6015718: ARHGAP40–[]–SLC32A1.* *The regional association plots show association test statistics versus position in the vicinity of the strongest associations. In the plots, a ‘+’ indicates an imputed variant, whereas a ‘x’ indicates an imputed protein-altering variant. Likewise, an ‘o’ symbol indicates a genotyped variant and a ‘◇’ (diamond) indicates a genotyped protein-altering variant. For each association, we also report details of the credible set, calculated under the assumption of a single causal variant within the locus.*

**Supplementary Figure 13**


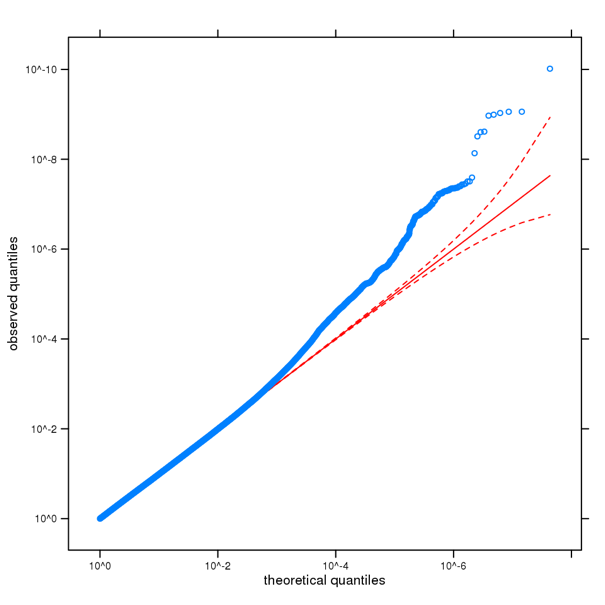


*Case (BD+MD) versus control European Q-Q plot of observed versus expected quantiles for the GWAS P values, where the expected distribution of P values is uniform under the null hypothesis, plotted on a log scale. A solid red line is shown with a slope of 1, and dashed red lines represent a 95% confidence envelope under the assumption that the test results are independent.*

**Supplementary Figure 14**

**
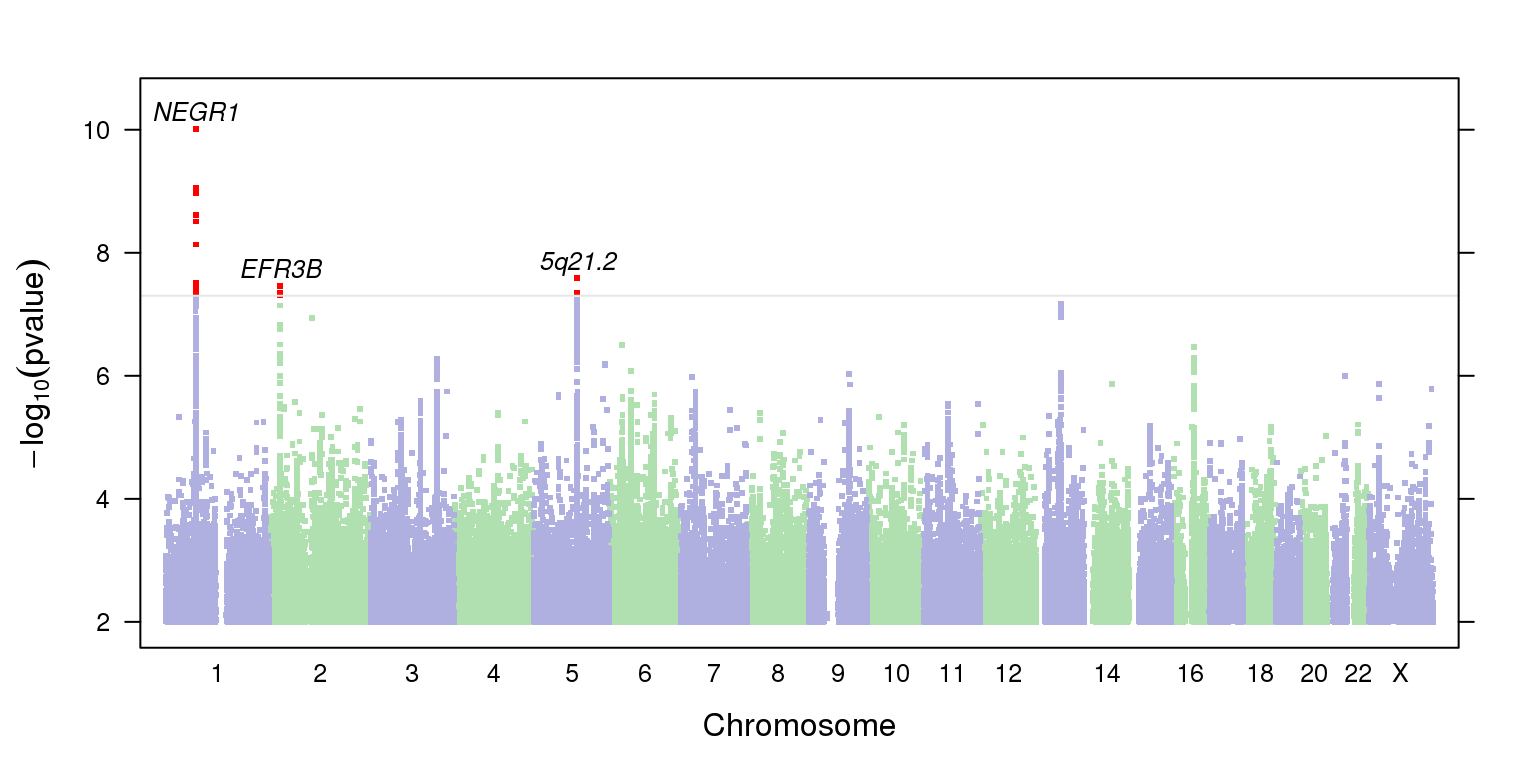
**

*Case (BD+MD) versus control European association results. Logistic regression assuming an additive model for allelic effects. Each SNP along the chromosome (x-axis) is plotted against the -log_10_(pvalue) test statistics. The results have been adjusted for a genomic control inflation factor****λ=1.073.*Supplementary Figure 15**


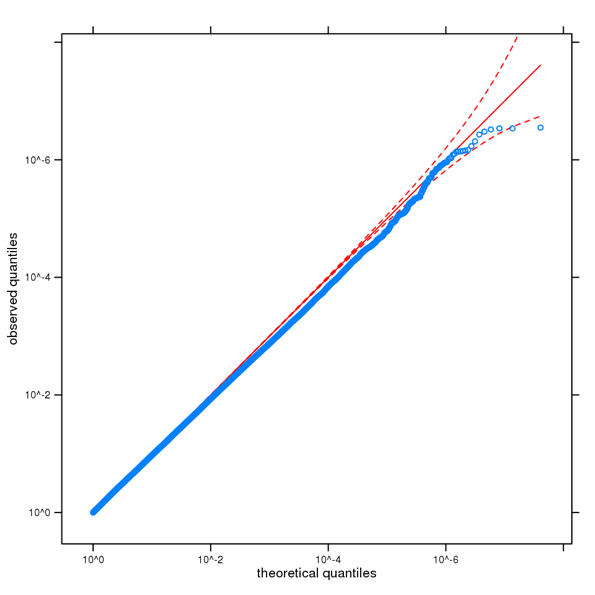


*BD versus MD European Q-Q plot of observed versus expected quantiles for the GWAS P values, where the expected distribution of P values is uniform under the null hypothesis, plotted on a log scale. A solid red line is shown with a slope of 1, and dashed red lines represent a 95% confidence envelope under the assumption that the test results are independent.*

**Supplementary Figure 16**


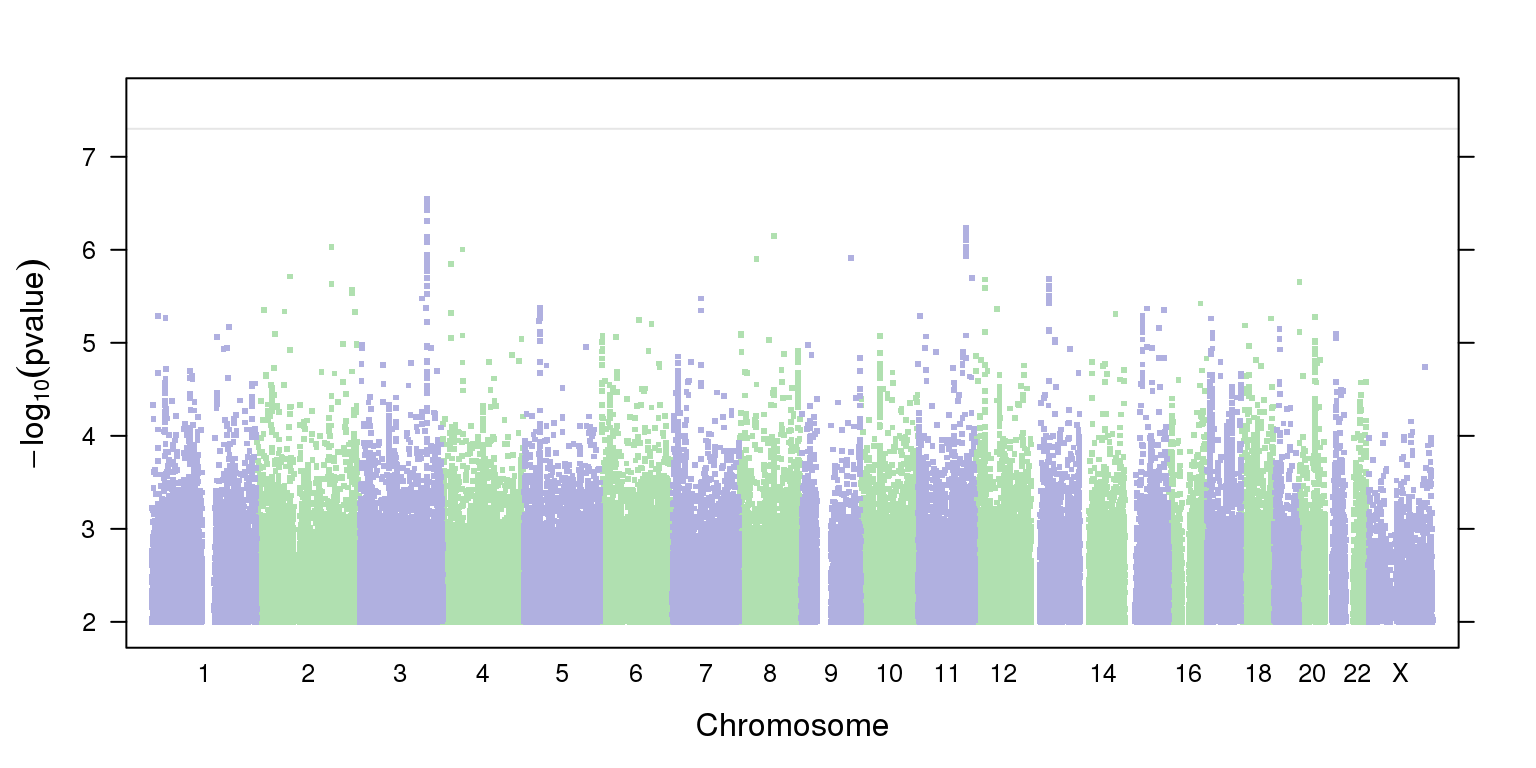


*BD versus MD European association Manhattan plot. Logistic regression assuming an additive model for allelic effects. Each SNP along the chromosome (x-axis) is plotted against the -log_10_(pvalue) test statistics. The results have been adjusted for a genomic control inflation factor****λ=1.048.***

**Supplementary Figure 17**


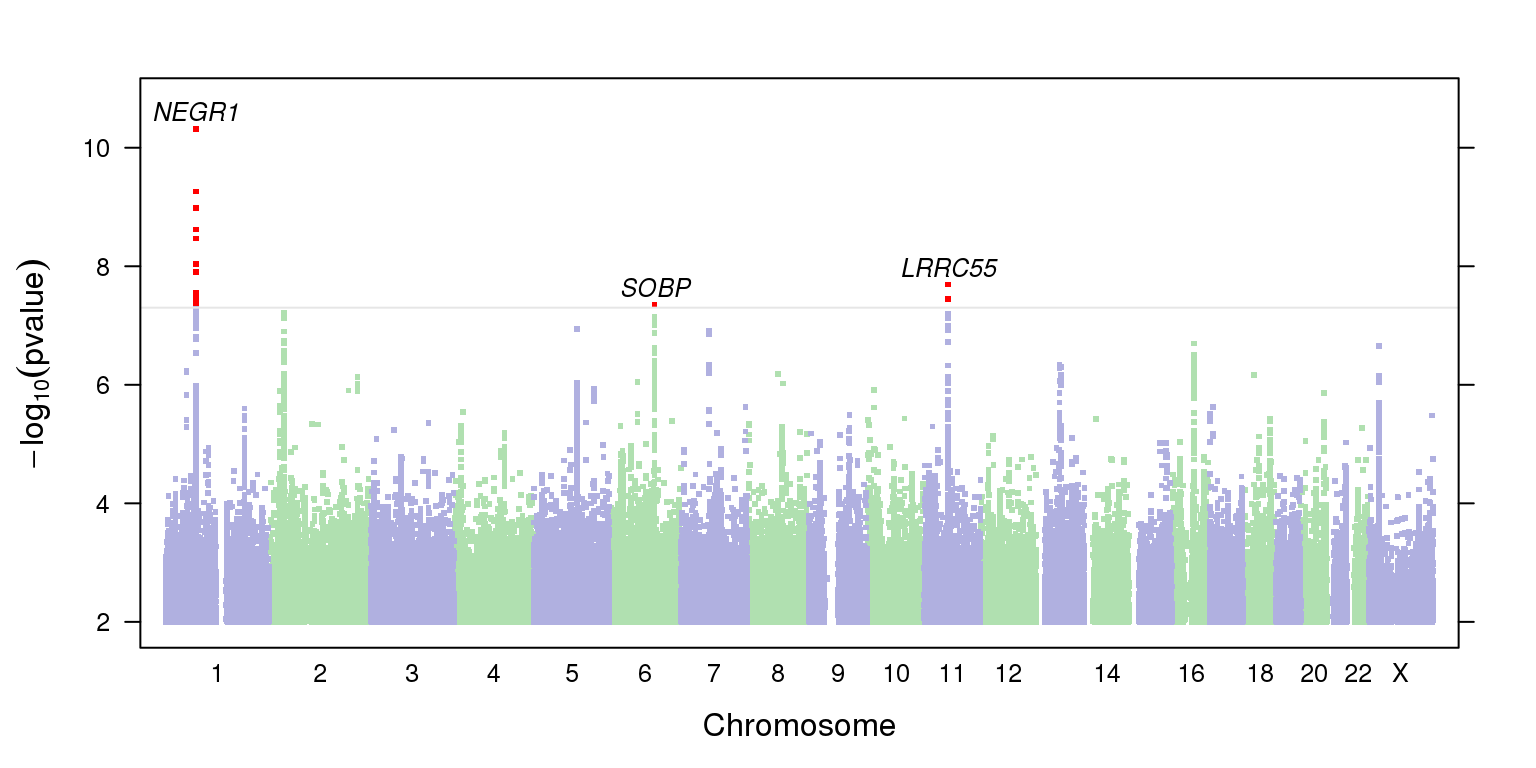


*MD versus control trans-ethnic association Manhattan plot.* *Logistic regression assuming an additive model for allelic effects. Each SNP along the chromosome (x-axis) is plotted against the -log_10_(pvalue) test statistics.*

**Supplementary Figure 18**

**A** rs12134600 **B** rs4946833


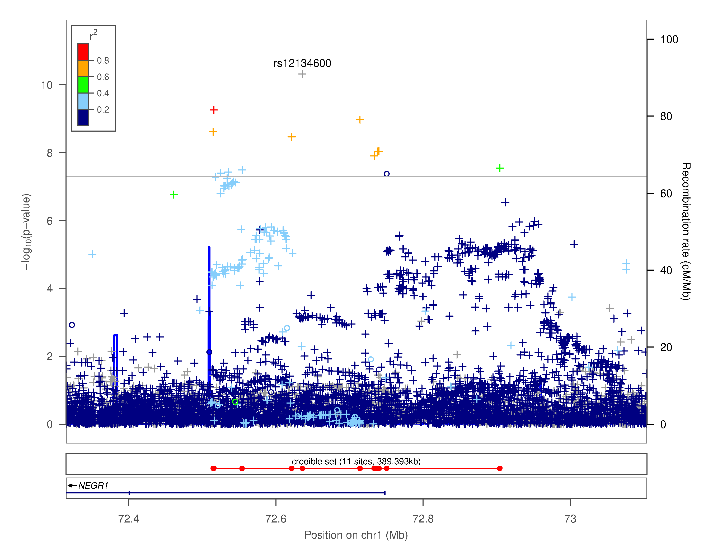

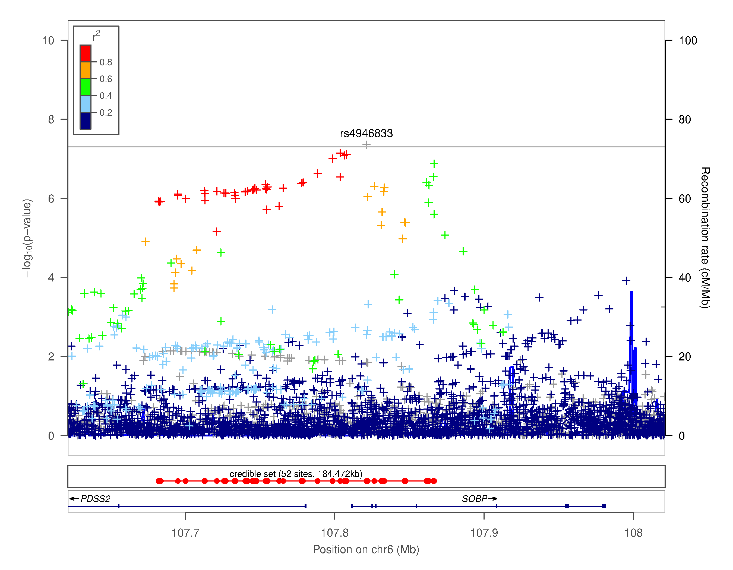


**C** rs11228927

**
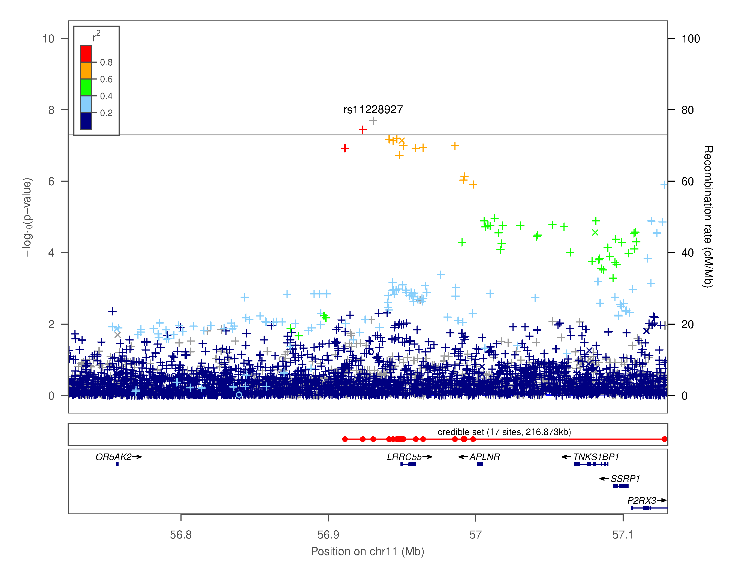
**

*MD versus control trans-ethnic GWAS meta-analysis regional plots for* ***A*** *rs12134600 - [NEGR1],* ***B*** *rs4946833 - [SOBP],* ***C*** *rs11228927 - OR5AK2—[]–LRRC55.* *The regional association plots show association test statistics versus position in the vicinity of the strongest associations. In the plots, a ‘+’ indicates an imputed variant, whereas a ‘x’ indicates an imputed protein-altering variant. Likewise, an ‘o’ symbol indicates a genotyped variant and a ‘◇’ (diamond) indicates a genotyped protein-altering variant. For each association, we also report details of the credible set, calculated under the assumption of a single causal variant within the locus.*

**Supplementary Figure 19**

**A** rs12134600 **B** rs4946833


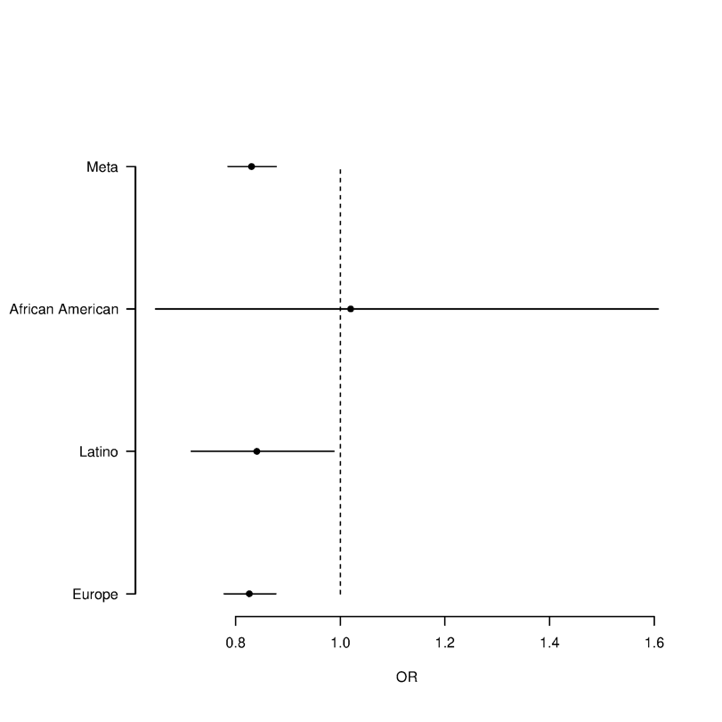

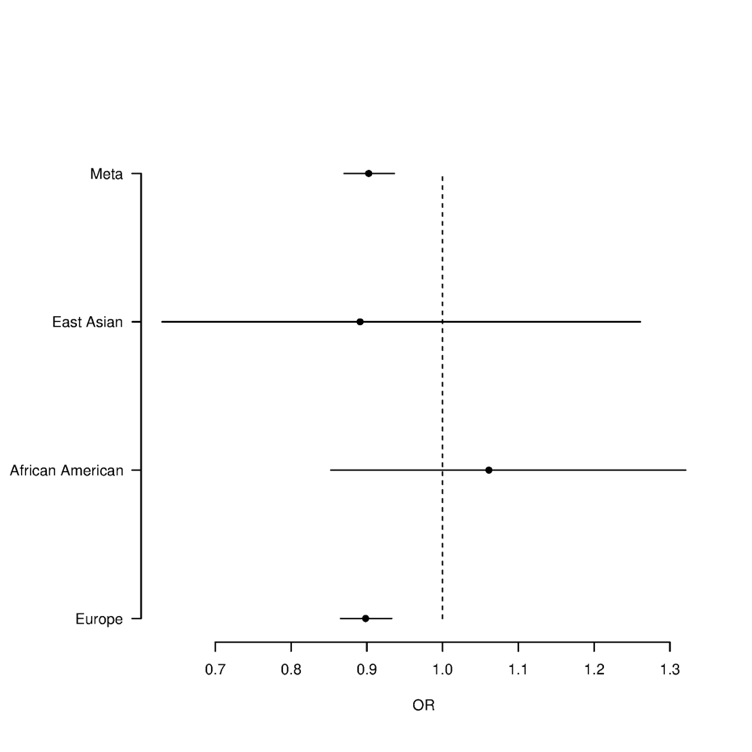


**C** rs11228927


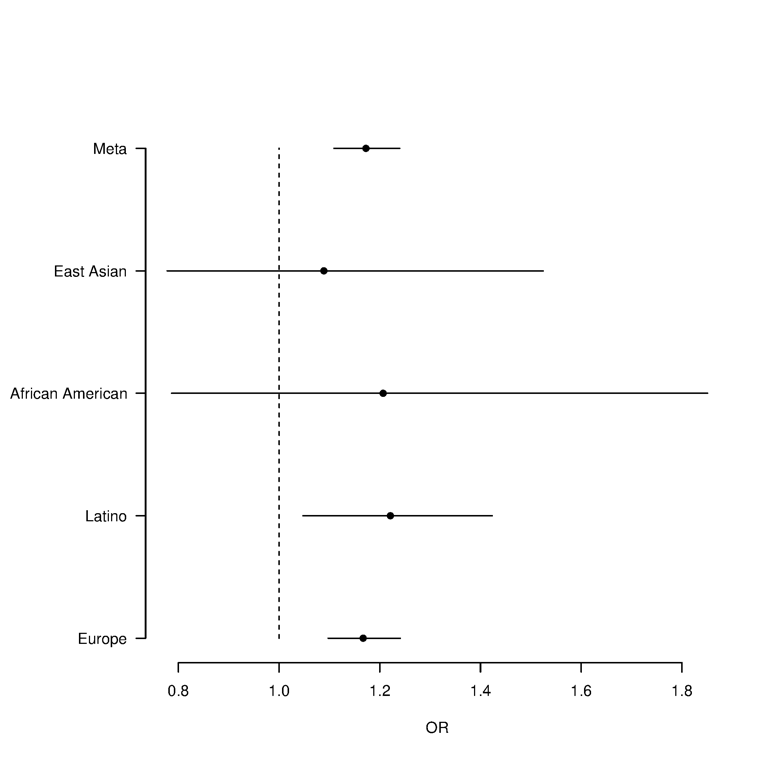


*MD versus control effect sizes from trans-ethnic GWAS meta-analysis effect sizes for* ***A*** *rs12134600 - [NEGR1],* ***B*** *rs4946833 - [SOBP],* ***C*** *rs11228927 - OR5AK2—[]–LRRC55. Effect sizes with 95% confidence intervals.*

**Supplementary Figure 20**


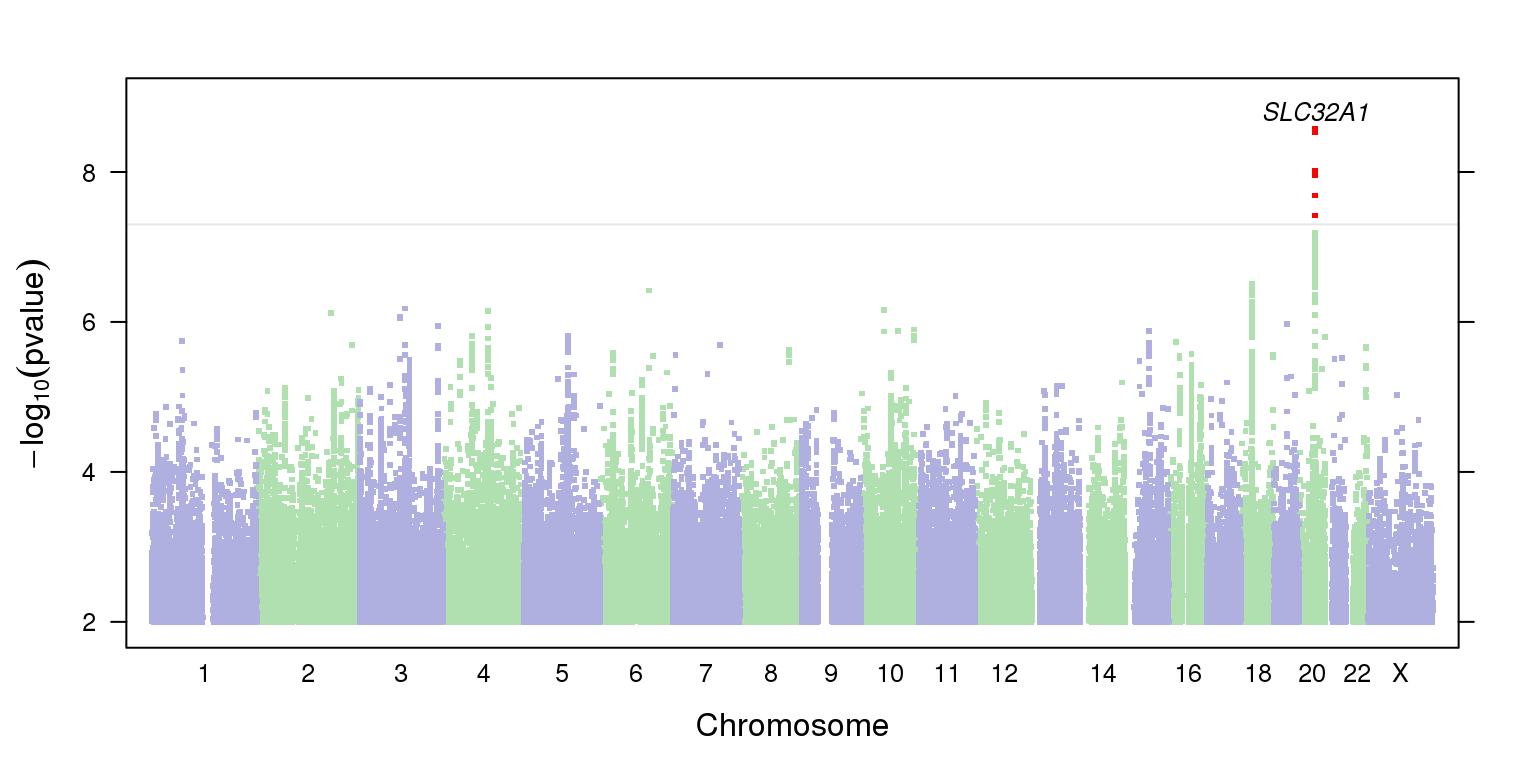


*BD versus control trans-ethnic association Manhattan plot.* *Logistic regression assuming an additive model for allelic effects. Each SNP along the chromosome (x-axis) is plotted against the -log_10_(pvalue) test statistics.*

**Supplementary Figure 21**


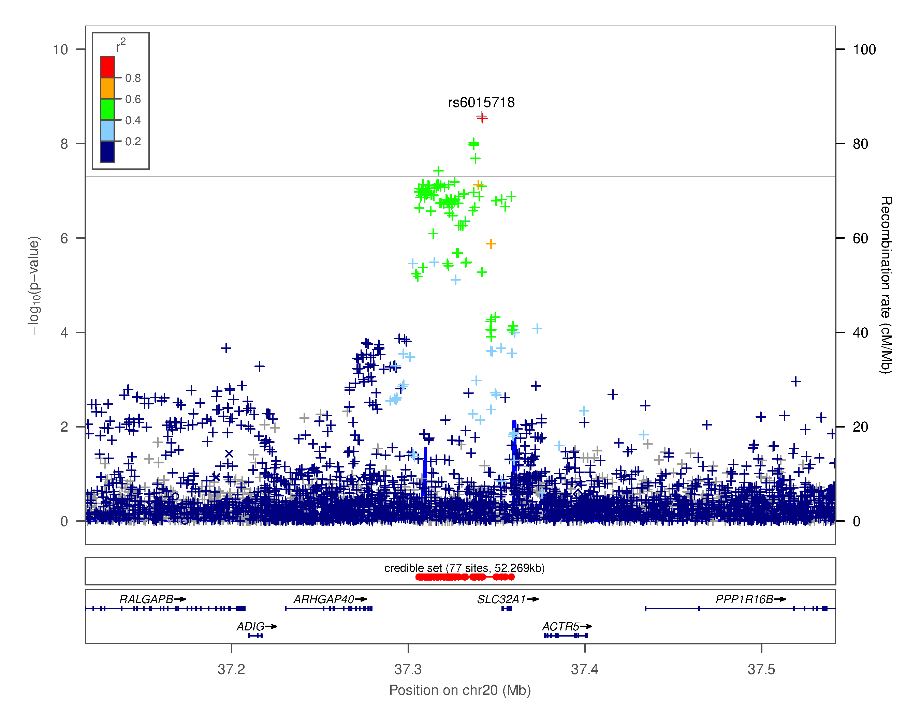


BD versus control trans-ethnic GWAS meta-analysis regional plots - rs6015718: ARHGAP40–[]–SLC32A1. *The regional association plots show association test statistics versus position in the vicinity of the strongest associations. In the plots, a ‘+’ indicates an imputed variant, whereas a ‘x’ indicates an imputed protein-altering variant. Likewise, an ‘o’ symbol indicates a genotyped variant and a ‘◇’ (diamond) indicates a genotyped protein-altering variant. For each association, we also report details of the credible set, calculated under the assumption of a single causal variant within the locus.*

**Supplementary Figure 22**

**
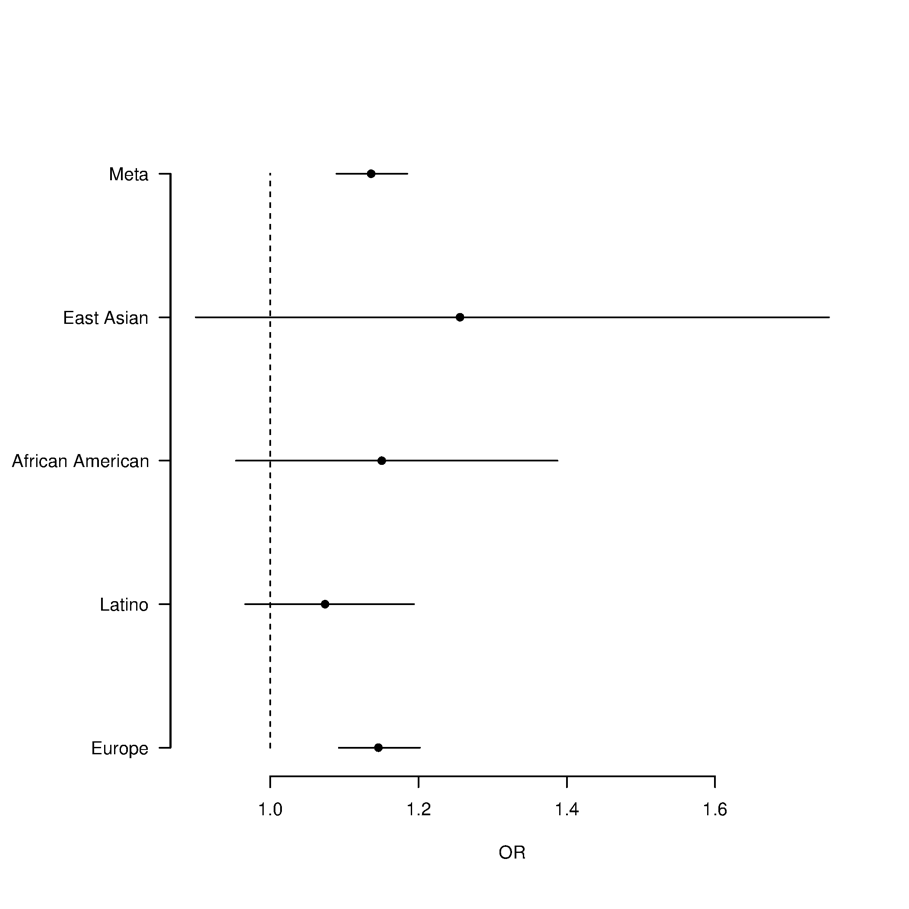
**

*BD versus control effect sizes for rs6015718: ARHGAP40–[]–SLC32A1 from trans-ethnic GWAS meta-analysis Effect sizes with 95% confidence intervals.*

**References**

1. Battle DE. Diagnostic and Statistical Manual of Mental Disorders (DSM). *CoDAS*. Published online 2013. doi:10.1007/978-3-642-28753-4_1094

2. Altman EG, Hedeker D, Peterson JL, Davis JM. The altman self-rating Mania scale. *Biol Psychiatry*. 1997;42(10):948-955. doi:10.1016/S0006-3223(96)00548-3

3. Altman E, Hedeker D, Peterson JL, Davis JM. A comparative evaluation of three self-rating scales for acute mania. *Biol Psychiatry*. 2001;50(6):468-471. doi:10.1016/S0006-3223(01)01065-4

4. Pilkonis PA, Choi SW, Reise SP, Stover AM, Riley WT, Cella D. Item banks for measuring emotional distress from the patient-reported outcomes measurement information system (PROMIS®): Depression, anxiety, and anger. *Assessment*. 2011;18(3):263-283. doi:10.1177/1073191111411667

5. Nolte S, Coon C, Hudgens S, Verdam MGE. Psychometric evaluation of the PROMIS® Depression Item Bank: an illustration of classical test theory methods. *J Patient-Reported Outcomes*. 2019;3(1):1-10. doi:10.1186/s41687-019-0127-0

6. Felitti VJ, Anda RF, Nordenberg D, Williamson DF, Spitz AM, Edwards V, et al. Relationship of childhood abuse and household dysfunction to many of the leading causes of death in adults: The adverse childhood experiences (ACE) study. *Am J Prev Med*. 1998;14(4):245-258. doi:10.1016/S0749-3797(98)00017-8

7. Browning SR, Browning BL. ARTICLE Rapid and Accurate Haplotype Phasing and Missing-Data Inference for Whole-Genome Association Studies By Use of Localized Haplotype Clustering. *Am J Hum Genet*. 2007;81:1084-1097. doi:10.1086/521987

8. Loh PR, Palamara PF, Price AL. Fast and accurate long-range phasing in a UK Biobank cohort. *Nat Genet*. 2016;48(7):811-816. doi:10.1038/ng.3571

9. Zheng X, Shen J, Cox C, Wakefield JC, Ehm MG, Nelson MR, et al. HIBAG - HLA genotype imputation with attribute bagging. *Pharmacogenomics J*. 2014;14(2):192-200. doi:10.1038/tpj.2013.18

10. Jia X, Han B, Onengut-Gumuscu S, Chen WM, Concannon PJ, Rich SS, et al. Imputing Amino Acid Polymorphisms in Human Leukocyte Antigens. Tang J, ed. *PLoS One*. 2013;8(6):e64683. doi:10.1371/journal.pone.0064683

11. Henn BM, Hon L, Macpherson JM, Eriksson N, Saxonov S, Pe’er I, et al. Cryptic distant relatives are common in both isolated and cosmopolitan genetic samples. *PLoS One*. 2012;7(4). doi:10.1371/journal.pone.0034267

12. Sullivan PF, Daly M, Ripke S, Lewis CM, Wray NR, Hamilton SP, et al. A mega-analysis of genome-wide association studies for major depressive disorder. *Mol Psychiatry*. 2013;18(4):497-511. doi:10.1038/mp.2012.21

13. Wray NR, Ripke S, Mattheisen M, Trzaskowski M, Byrne EM, Abdellaoui A, et al. Genome-wide association analyses identify 44 risk variants and refine the genetic architecture of major depression. *Nat Genet*. 2018;50(5):668-681. doi:10.1038/s41588-018-0090-3

14. Howard DM, Adams MJ, Clarke TK, Hafferty JD, Gibson J, Shirali M, et al. Genome-wide meta-analysis of depression identifies 102 independent variants and highlights the importance of the prefrontal brain regions. *Nat Neurosci*. 2019;22(3):343-352. doi:10.1038/s41593-018-0326-7

15. Hyde CL, Nagle MW, Tian C, Chen X, Paciga SA, Wendland JR, et al. Identification of 15 genetic loci associated with risk of major depression in individuals of European descent. *Nat Genet*. 2016;48(9):1031-1036. doi:10.1038/ng.3623

16. Stahl EA, Breen G, Forstner AJ, McQuillin A, Ripke S, Trubetskoy V, et al. Genome-wide association study identifies 30 loci associated with bipolar disorder. *Nat Genet*. 2019;51(5):793-803. doi:10.1038/s41588-019-0397-8

17. Mullins N, Forstner AJ, O KS, Sloofman LG, Steinberg S, Trubetskoy V, et al. Genome-wide association study of over 40,000 bipolar disorder cases provides novel biological insights. *medRxiv*. 2020;17(13):202. https://doi.org/10.1101/2020.09.17.20187054

18. Ripke S, Neale BM, Corvin A, Walters JTR, Farh KH, Holmans PA, et al. Biological insights from 108 schizophrenia-associated genetic loci. *Nature*. 2014;511(7510):421-427. doi:10.1038/nature13595

19. Schizophrenia Working Group of the Psychiatric Genomics Consortium., Ripke S, Walters JT, O’Donovan MC. Mapping genomic loci prioritises genes and implicates synaptic biology in schizophrenia. *medRxiv*. Published online 2020:2020.09.12.20192922. https://doi.org/10.1101/2020.09.12.20192922
